# Aeroallergens and Climate Change in Tulsa, Oklahoma: Long-Term Trends in the South Central United States

**DOI:** 10.3389/falgy.2021.726445

**Published:** 2021-10-07

**Authors:** Estelle Levetin

**Affiliations:** Department of Biological Science, University of Tulsa, Tulsa, OK, United States

**Keywords:** climate change, aeroallergens, aerobiology, *Ambrosia* pollen, Cupressaceae pollen, long-term pollen monitoring

## Abstract

Climate change is having a significant effect on many allergenic plants resulting in increased pollen production and shifts in plant phenology. Although these effects have been well-studied in some areas of the world, few studies have focused on long-term changes in allergenic pollen in the South Central United States. This study examined airborne pollen, temperature, and precipitation in Tulsa, Oklahoma over 25 to 34 years. Pollen was monitored with a Hirst-type spore trap on the roof of a building at the University of Tulsa and meteorology data were obtained from the National Weather Service. Changes in total pollen intensity were examined along with detailed analyses of the eight most abundant pollen types in the Tulsa atmosphere. In addition to pollen intensity, changes in pollen season start date, end date, peak date and season duration were also analyzed. Results show a trend to increasing temperatures with a significant increase in annual maximum temperature. There was a non-significant trend toward increasing total pollen and a significant increase in tree pollen over time. Several individual taxa showed significant increases in pollen intensity over the study period including spring Cupressaceae and *Quercus* pollen, while *Ambrosia* pollen showed a significant decrease. Data from the current study also indicated that the pollen season started earlier for spring pollinating trees and Poaceae. Significant correlations with preseason temperature may explain the earlier pollen season start dates along with a trend toward increasing March temperatures. More research is needed to understand the global impact of climate change on allergenic species, especially from other regions that have not been studied.

## Introduction

Over the past two decades, numerous studies and reviews have shown that increasing atmospheric CO_2_ levels and global warming have resulted in enhanced plant growth and reproduction as well as changes to plant phenology with earlier flowering and longer growing seasons (1–6). While the changes in productivity may be tied to the enrichment of CO_2_ on rates of photosynthesis, the changes in plant phenology are related to warmer winter and spring temperatures and delayed frosts in autumn.

Many scientific papers have addressed the effects of climate change on plants known to produce allergenic pollen. Warmer temperatures in many areas of the northern hemisphere have led to earlier spring pollen seasons, increased pollen intensity, and longer pollen seasons along with more suffering among those with pollen allergies and asthma (4, 7–13). However, not all areas and not all plant species have been affected by climate change. Pollen data from three cities in southern Spain suggest that from 1994 to 2017, pollen season start dates were delayed in some cities for *Ulmus, Alnus* and *Populus* (14). In addition to effects on late winter and spring-pollinating plants, studies have shown longer *Ambrosia* pollen seasons in the fall (15–17). Ziska et al. (15) showed that the *Ambrosia* pollen season in North America lengthened from 1995 to 2009 due to the delay of the first frost in northern areas. This outcome was most evident in Winnipeg and Saskatoon, the two monitoring sites in Canada, where the pollen season was 25 and 27 days longer, respectively. Investigations have also found that various allergenic taxa have increased biomass, produced more flowers and more pollen under experimentally elevated CO_2_ levels. Among the taxa studied have been *Ambrosia* (18–20), *Pinus* (21), *Phleum* (22), and *Betula* (23).

Climate change can alter the distribution of many plant taxa. These landscape changes are due to latitudinal or altitudinal shifts, range expansion or reduction, or the invasion of alien species (3, 4, 24, 25). In a meta-analysis of long-term data, Parmesan and Hanley (4) concluded that major shifts in the distribution of plant species have already occurred. For example, warming temperatures have contributed to the expansion of *Ambrosia artemisiifolia* in Europe (26–32). This species has become invasive in many areas of Eastern and Central Europe over several decades and is now a leading health issue in many countries. Several studies have used computer models to predict the future expansion of *Ambrosia* in Europe and the resulting increase in pollen sensitization (28–32). The simulations also suggest that *Ambrosia* pollen concentrations will be higher and the pollen season will be longer (31). Similar spread of *A. artemisiifolia* has been documented on other continents as well (33).

In many areas throughout the world, woody plant encroachment or invasion into grasslands and savannas has been occurring for decades (34–37). Various explanations have been proposed to account for this expansion including fire suppression, overgrazing by livestock, increasing CO_2_ levels, and local climate (34–37). In the Great Plains of North America, the most notable landscape change has been the encroachment of *Juniperus virginiana* L. into the prairie. Although *J. virginiana* is native to Oklahoma, the expansion of this species has been well-documented in recent decades (36–40). Similar expansion of *J. ashei* Buchholz has also been described in south central Oklahoma and central Texas (40, 41). Using images acquired by satellites, Wang et al. (40) estimated that *Juniperus* woodlands have replaced Oklahoma grasslands on over 130,000 hectares from 1984 to 2010. They also indicated that the expansion continues at a rate of around 4,800 hectares per year. In addition, *J. virginiana* has also increased its distribution within the Cross Timbers ecotone of the southern Great Plains found in Texas, Oklahoma, and Kansas (42–45). The Cross Timbers is a transition zone between the eastern deciduous forests and the prairie and is characteristically dominated by *Quercus stellata* Wangenh. and *Q. marilandica* Muenchh. The encroachment of *J. virginiana* is transforming the Cross Timbers into a closed canopy oak/juniper forest.

Very few studies have addressed the influence of climate change on allergenic pollen in the South Central United States. Zhang et al. (10) examined pollen season variations in the United States from 1994 to 2010 for *Ambrosia, Artemisia, Betula*, Poaceae, and *Quercus* pollen using data from 50 stations of the National Allergy Bureau (NAB), the aeroallergen monitoring network from the American Academy of Allergy, Asthma, and Immunology. Data from eight NAB stations were compiled to represent the South (south central states). For the combined data for the 50 stations in the United States, the study showed earlier pollen season start, increased pollen intensity, and increased peak concentrations for the period 2001 to 2010 as compared to 1994–2000. However, for the combined data for the South and Southeast, the authors found later pollen season onset and shorter season duration for the same periods. Anderegg et al. (11) examined pollen trends in North America from 1990 to 2018 from 57 NAB stations plus three additional pollen monitoring stations not affiliated with the NAB. Twelve NAB stations in the South Central United States were included in the study. Data showed increased spring and annual pollen concentrations and longer seasons for spring pollen across North America. The authors found that climate change was the central factor driving the increase in spring pollen season length and contributed to the increase in pollen intensity. By contrast, monthly pollen integrals for summer and fall pollen seasons showed decreases with the decrease significant for June, July, and August.

Although not directly addressing climate change, previous studies from the Levetin lab have examined some long-term trends in aeroallergens. Levetin (46) examined the yearly variation in pollen season metrics from 1987 to 1996 for several late winter and early spring taxa. The data showed a significant increase in SPIn for Cupressaceae pollen and non-significant increases for *Quercus* and *Ulmus*. Howard and Levetin (47) examined the aerobiology of *Ambrosia* pollen from 1987 to 2013 for the development of a forecasting model for daily exposure. In looking at long term trends, they showed significant decreases over time in the SPIn for *Ambrosia* pollen, but no significant changes in start date, end date, season duration, or peak date. In a study on the aerobiology of *J. virginiana* pollen from 1987 to 2016, Flonard et al. (48) showed significant increases over time in the pollen intensity, which paralleled the species expansion in Oklahoma. The aim of the current study was to examine the influence of global warming on the airborne pollen levels in Tulsa, Oklahoma from 1987 to 2020.

## Materials and Methods

### Study Site

**Figure 1:**
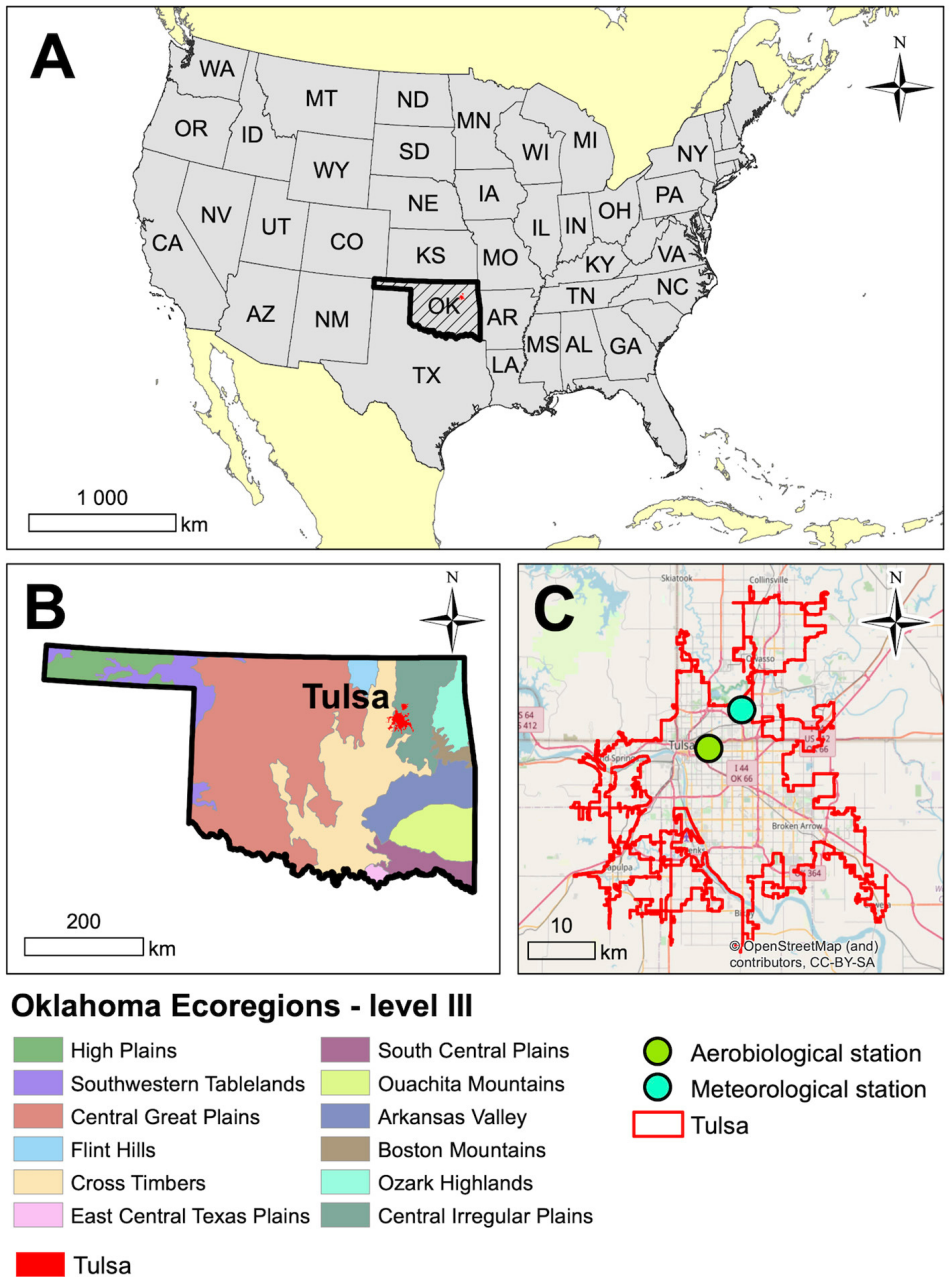
Location of Tulsa, Oklahoma in the South-Central United States. **(A)** Location of Oklahoma in the USA, **(B)** Tulsa in Oklahoma on the background of ecoregions, and **(C)** Aerobiological and meteorological stations in Tulsa.

This study was carried out in Tulsa, located in northeast Oklahoma in the South Central United States (Figure 1). Oklahoma is in a biological transition zone within the continent ranging from the deciduous forests in the east to the short grass prairie of western Oklahoma (49, 50). As a result, Oklahoma has a large, well-defined floral diversity, which is also evident in Tulsa area. Although portions of Tulsa lie in the deciduous forest biome, the eastern part of the city lies in a segment of tallgrass prairie biome that extends south from Kansas.

Tulsa has a continental climate and generally has mild winters and long, hot summers. The average yearly precipitation in Tulsa is 1,038 mm (51) and the mean yearly temperature is 16.25 C. July is the hottest month with a mean temperature of 28.54 C and January the coldest with the mean of 3.58 C. Temperature and precipitation means are 30 year averages. Prevailing winds are from the south bringing warm, moist air from the Gulf of Mexico; however, northerly winds are also common and generally associated with the passage of frontal systems.

### Air Sampling

The atmosphere in Tulsa, Oklahoma has been monitored with a Hirst-type spore trap (Burkard Manufacturing, Rickmansworth, UK) since December 1986. The sampler is located on the roof of the Biological Science building (Longitude −95.9454, Latitude 36.1511) at the University of Tulsa, at a height of 12 meters above ground. From 5 Jul to 31 Aug 1994, data from a second Burkard sampler was used while reroofing of Biological Science building was on-going. The second sampler was located in a residential area approximately 6 km southwest of campus. Sampler operation followed standard methods used throughout the study period (46–48) and identical to guidelines recommended by the Pan American Aerobiology Association (52). Over the 34 years, Burkard slides were analyzed by several different individuals using different methods including one (3.4% of the slide surface), two (6.9% of the slide surface), three (10.3% of the slide surface), or four (13.6% of the slide surface) longitudinal traverses or 12 transverse traverses (11.5% of the slide surface). For several years more than one method was used and the results averaged for the database. Analysis of the *Ambrosia* pollen data from our lab found highly significant correlations (r > 0.95, *p* < 0.001) between concentrations determined by different counting methods, and the methods were considered roughly synonymous in estimates of the daily concentrations (47). Similar results were also obtained for correlations of other pollen types (Cupressaceae, *Morus*, Poaceae, and *Quercus*) which were analyzed by different counting methods.

### Pollen Data

From January 1987 through December 1995, only certain pollen taxa were being investigated; as a result, data for the following pollen types were available starting from January 1987: *Ambrosia, Betula, Carya*, Cupressaceae, *Morus/Broussonetia*, and *Ulmus*. Poaceae and *Quercus* pollen data were incomplete for 1987, so the analyses for these taxa are considered from 1988. Since 1996, data have been regularly collected for 41 pollen types (Supplementary Table 1). Nine of the pollen taxa were registered in low numbers and only occurred during a few years. For Supplementary Table 1, these were included in the category “Other Pollen” along with unknown and indeterminant pollen grains.

Temporal analysis of total airborne pollen levels was analyzed using the 1996 to 2020 dataset. Additional detailed analyses focused on the eight most abundant pollen taxa in the Tulsa atmosphere; these include *Ambrosia, Celtis*, Cupressaceae, *Morus/Broussonetia, Platanus*, Poaceae, *Quercus*, and *Ulmus*. Collectively, these taxa account for more than 83% of the pollen registered from 1996 through 2020 and include the major airborne allergens in the area. The analyses of these individual taxa utilized all the years of data available for each taxon.

Pollen season metrics for the eight taxa include four pollen season timing variables: the pollen season start date, end date, duration (number of days from start date to end date), and peak date. Other metrics assess pollen intensity and include peak concentration and the annual or seasonal pollen integral (APIn or SPIn). The APIn is the sum of the average daily pollen concentration for the whole year, while the SPIn is the sum of the average daily pollen concentration for a defined period (53).

The Cupressaceae and *Ulmus* pollen captured in the Tulsa atmosphere each represent two or more distinct pollen seasons. Cupressaceae pollen occurs in the Tulsa atmosphere for 9 months and represents three distinct seasons, referred to as *spring, fall*, and *winter* Cupressaceae. The spring Cupressaceae season (February through May) primarily represents pollen from native *Juniperus virginiana* as well as various ornamentals in the family that are used in landscaping (48). During the fall (September through November) pollen from *J. pinchotii* Sudw is registered in Tulsa, resulting from long-distance transport from southwestern Oklahoma and west Texas (54, 55). In winter (December and January), long-distance transport events carry pollen from *J. ashei* woodlands in south central Oklahoma and central Texas to Tulsa (55–61). The data for these seasons were analyzed separately. Pollen integrals for these taxa were computed for each pollinating season not for the calendar year. For the spring Cupressaceae, the SPIn was the sum of the average daily concentration from 1 February to 31 May. For *J. pinchotii* the SPIn was the sum from 1 September to 30 November and for *J. ashei* from 1 December to 31 January. The pollen curves of the three seasons are shown in Supplementary Figure 1. These dates are supported by field work in the Tulsa area for *J. virginiana* as well as in south central and southwestern Oklahoma and Texas for the other species. No open cones could be found in Tulsa before February. In Texas by late January most *J. ashei* pollen cones had either abscised or were empty, and few cones were mature in November. For *J. pinchotii* most cones were empty or abscised in late November. In addition, Mohanty et al. (55) used quantitative PCR to identify the species of Cupressaceae pollen registered in our Tulsa air samplers from October 2013 to April 2015. During the two seasons, there were a total of 8 days in January and February when both *J. ashei* and *J. virginiana* pollen were detected in the Tulsa air samples. On six of those days, *J. virginiana* pollen was detected in January with an average concentration of 6 pollen grains/m^3^ and on 2 days *J. ashei* was detected in February with an average concentration of 1 pollen grain/m^3^. Similar results were found in the fall with a total of 5 days with both *J. ashei* and *J. pinchotii* registered in the Tulsa air samples. On 4 days *J. ashei* pollen was detected in November with an average concentration of <1 pollen grain/m^3^, and on 1 day in December *J. pinchotii* pollen was detected with a concentration of <1 pollen grain/m^3^.

The *Ulmus* pollen registered in Tulsa represents two distinct seasons referred to as *spring* and *fall*. The spring *Ulmus* pollen season occurs from February through April and represents pollen from *Ulmus americana* L., *U. alata* Michx., *U. pumila* L., and *U. rubra* Muhl (49, 62). The fall *Ulmus* season occurs during late summer through fall (July through October) when three additional *Ulmus* species are in flower, *U. crassifolia* Nutt., *U. parvifolia* Jacq., and *U. serotina* Sarg (49, 62). While pollen data for the spring *Ulmus* season were analyzed since 1987, data for the fall *Ulmus* season were counted beginning in 1996. The data for the two *Ulmus* seasons were analyzed separately and the pollen integrals were computed for the separate seasons. The average daily pollen concentration was summed from 1 January to 30 June for the spring *Ulmus* season SPIn and from the 1 July to 31 December for the fall *Ulmus* season SPIn. The average daily concentrations of the spring and fall season are shown in Supplementary Figure 1.

Research has shown that there is no single method for determining the pollen season start date for all taxa (63–68). As a result, several methods (63–65, 67, 69) were utilized, and the method that corresponded best to the pollen data was chosen for each pollen type. The method selected for each pollen type was used for all years for that taxon.

The methods proposed by Pfaar et al. (67) were chosen for *Celtis*, Cupressaceae, *Morus/Broussonetia, Platanus*, Poaceae, *Quercus*, and the spring *Ulmus* pollen seasons. For the tree pollen taxa, the season started on the first day of 5 days (in 1 week) when the pollen concentration on each of the 5 days was ≥10 pollen grains/m^3^ and the sum of the 5 days was at least 100. The season end was considered as the last day of 5 days that met these same conditions. For Poaceae pollen, the season started on the first day of 5 days (in 1 week) when the pollen concentration on each of the 5 days was ≥3 pollen grains/m^3^ and the sum of the 5 days was at least 30. The end of the season was considered the last day meeting the same criteria.

The Pfaar method was not suitable for *Ambrosia* pollen or for the fall *Ulmus* season. For *Ambrosia* pollen, the Pfaar method showed end dates late in the year, generally after local plants had senesced, and in several years, the Pfaar end date was after a frost that was equal to or below −2.0°C. The 98% method (69) recognizes the season start at 1% of the APIn and season end at 99% of the APIn. This method was used for the *Ambrosia* pollen data; however, the calculations were based on the cumulative pollen level registered from 1 August until the date of the first frost which was −2.0°C or lower. This was to prevent the inclusion of resuspended pollen or pollen from long range transport. For the fall *Ulmus* pollen season, registered pollen occurred in short spikes of a few days followed by days without *Ulmus* pollen. No start date could be determined during 8 years using the Pfaar method. The 90% method (63) was chosen for the late *Ulmus* pollen season. This method designates the season start at 5% of the APIn or SPIn and season end at 95%. The use of the 90% method eliminated some of early spikes with the low pollen concentrations at the beginning of the season.

### Meteorological Data

Meteorological data from the National Weather Service office in Tulsa were used in this study; the Tulsa office is approximately 8 km northeast of the air sampling station on campus. Maximum (T-max) and minimum (T-min) daily temperature values were used to calculate daily means as well as monthly, seasonal, and yearly averages. Seasonal averages were based on meteorological seasons (70). Averages for spring were calculated from March, April, and May data, for summer from June, July and August data, and for fall from September, October, and November data. For winter the seasonal averages included January and February data along with data from December of the previous year. Daily precipitation data from the National Weather Service station in Tulsa were used to determine total monthly and yearly precipitation.

### Data Analysis

Microsoft Excel 2018 was used to compile and analyze the pollen metrics and meteorological data as well as for the Mann-Kendal analysis. Statistica (10.0) was used for additional analyses. Simple linear regression was used to analyze pollen metrics and temperature values over time and Pearson correlation was used to analyze the pollen season timing variables with temperature variables since these variables met the normality requirements for Pearson correlation. Spearman correlation was used to analyze peak pollen concentrations and APIns or SPIns with monthly temperature variables and for all correlations with monthly precipitation. Mann–Kendall test for the trend analysis with Sen's slope estimator was used for the analysis of precipitation data since data were not normally distributed.

## Results

### Temperature and Precipitation Trends

**Figure 2:**
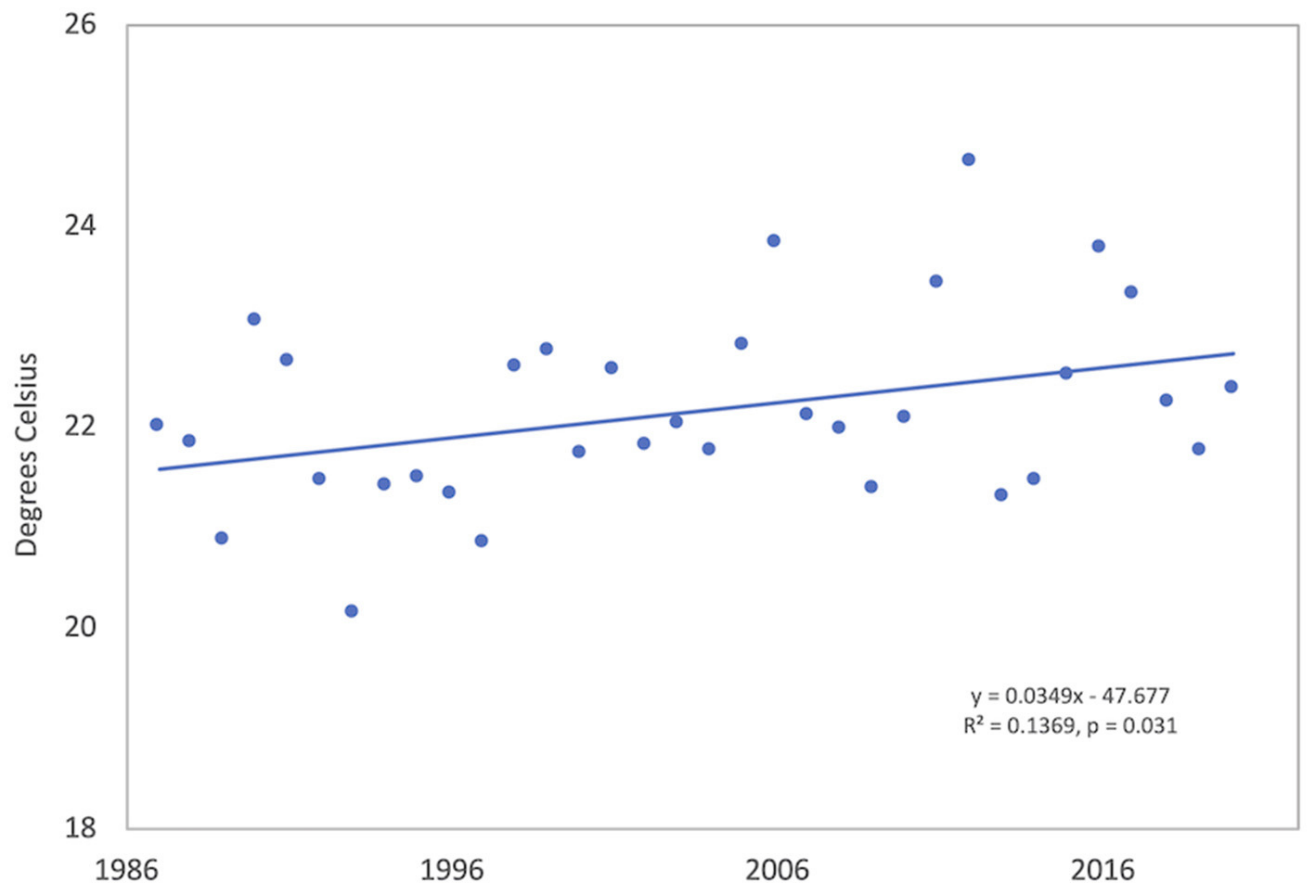
Annual maximum air temperature (T-max) in Tulsa, Oklahoma from 1987 to 2020. Meteorological data are from the Tulsa National Weather Service station approximately 8 km from the air sampling site.

The mean annual temperature in Tulsa, Oklahoma was 16.4°C during the study period; the mean T-max was 22.2°C and the mean T-min 10.4°C. The hottest year was 2012 and the coldest was 1993 (Supplementary Table 2). In general, yearly temperatures were quite variable; however, linear regression of yearly temperatures from 1987 to 2020 showed trends toward increasing temperatures. Yearly T-max significantly increased over time (R^2^ = 0.137, *p* = 0.031) with a temperature change of 1.19 °C during 34 years (Table 1, Figure 2). The positive trends in T-min (R^2^ = 0.029, *p* = 0.332) and T-mean (R^2^ = 0.070, *p* = 0.130) were not significant.

**Table 1:** Linear regression analysis of yearly, seasonal, and monthly maximum and minimum temperatures during 1987–2020.

**Maximum Temperature**					**Minimum Temperature**				
	***R*** ^**2**^	**Slope**	***p***	**Δ°** **C**		***R*** ^**2**^	**Slope**	***p***	**Δ°** **C**
**Yearly Means**									
T-max	**0.137**	**0.0349**	**0.031**	**1.19**	T-min	0.029	0.0125	0.332	0.42
**Seasonal Means**									
Winter	0.035	0.032	0.286	1.10	Winter	0.004	−0.009	0.722	−0.29
Spring	0.049	0.029	0.207	0.97	Spring	0.014	0.015	0.502	0.50
Summer	0.045	0.030	0.231	1.02	Summer	0.047	0.020	0.220	0.68
**Fall**	**0.129**	**0.048**	**0.037**	**1.64**	Fall	0.041	0.022	0.252	0.76
**Monthly Means**									
January	0.043	0.050	0.237	1.70	January	0.005	−0.012	0.687	−0.41
February	0.000	−0.005	0.920	−0.18	February	0.012	−0.026	0.540	−0.88
March	0.087	0.068	0.090	2.33	March	0.043	0.047	0.239	1.59
April	0.003	0.009	0.768	0.30	April	0.000	0.004	0.902	0.14
May	0.002	0.008	0.804	0.26	May	0.003	−0.007	0.776	−0.24
June	0.113	0.059	0.052	2.02	**June**	**0.144**	**0.050**	**0.027**	**1.70**
July	0.054	0.041	0.186	1.40	July	0.013	0.015	0.522	0.51
August	0.002	−0.009	0.792	−0.31	August	0.001	−0.004	0.879	−0.12
**September**	**0.119**	**0.069**	**0.045**	**2.36**	**September**	**0.116**	**0.063**	**0.049**	**2.14**
October	0.011	0.019	0.561	0.65	October	0.011	0.016	0.555	0.54
November	0.051	0.057	0.200	1.94	November	0.003	−0.012	0.747	−0.40
December	0.049	0.051	0.210	1.72	December	0.004	0.014	0.710	0.48

*Temperature change (Δ°C) over time based on these analyses are also indicated. Statistically significant trends are in bold face*.

Monthly and seasonal T-max and T-min temperatures were also analyzed by linear regression (Table 1). September T-max showed the only significant increase in T-max (R^2^ = 0.119, *p* = 0.045) with an increase of 2.36°C during 34 years. T-Max also showed over 2°C increases during March and June but these were not significant. T-min in June (R^2^ = 0.1440, *p* = 0.027) and September (R^2^ = 0.116, *p* = 0.049) had significant increases during this period with changes of 1.70 and 2.14°C, respectively. Results of seasonal analyses found that only fall (September through November) T-max had a significant increase over time of 1.64°C (R^2^ = 0.129, *p* = 0.037).

The mean yearly precipitation during the study period was 1,030 mm. Precipitation was highly variable with the lowest yearly total at 716 mm in 2016 and the highest at 1,569 mm in 2015 (Supplementary Table 2). Analysis of the yearly and monthly total precipitation over time showed no significant trends (Table 2).

**Table 2:** Mann–Kendall test for trend analysis with Sen's slope estimator for total yearly and monthly precipitation during the period 1987–2020.

	**Z Statistic**	**p**	**Sen's Slope**
Yearly Precipitation	0.148	ns	0.610
**Monthly Precipitation**			
January	−0.400	ns	−0.218
February	−0.222	ns	−0.095
March	0.638	ns	0.554
April	0.949	ns	1.157
May	0.400	ns	0.487
June	−0.089	ns	−0.183
July	1.171	ns	0.742
August	1.186	ns	1.306
September	−1.646	ns	−1.251
October	1.497	ns	1.330
November	−0.504	ns	−0.365
December	−0.875	ns	−0.693

### Total Airborne Pollen From 1996 to 2020

**Figure 3:**
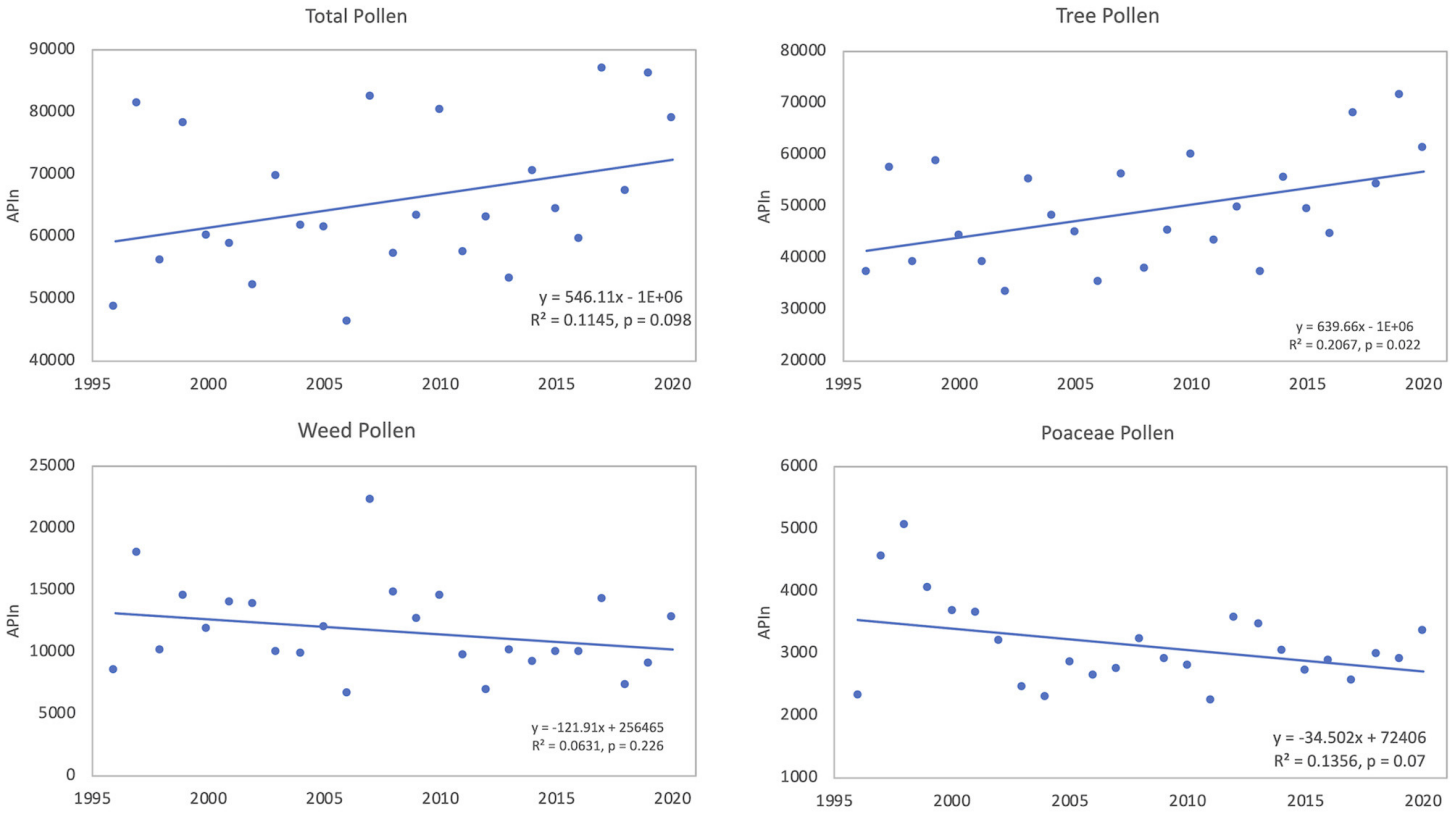
Linear trends of APIn from 1996 to 2020 for total pollen, tree pollen taxa, weed pollen taxa, and Poaceae pollen.

During the period of 1996 to 2020 all pollen types were counted and data from this period was used to look at trends in total pollen intensity as well as intensity of tree pollen, weed pollen, and Poaceae pollen. APIn of total pollen was variable and ranged from a low of 46,282 in 2006 to 86,902 in 2017; linear regression was used to analyze for any changes of total airborne pollen. Analysis showed that during the 25 years, the APIn for total pollen increased (Figure 3); however, the regression results were not significant (R^2^ = 0.115, *p* = 0.098). Pollen data were categorized into tree, weed and Poaceae pollen with tree pollen representing 74.6%, weed pollen 17.7%, and Poaceae pollen 4.7% of the total pollen during this period. The APIn of total tree pollen showed significant increases over time (R^2^ = 0.207, *p* = 0.022) (Figure 3). There were decreases in the APIn for both Poaceae and weed pollen, but neither trend was significant (Figure 3).

### Individual Pollen Taxa

The eight most abundant pollen taxa in the Tulsa atmosphere were selected for detailed analyses of the pollen season timing variables and the pollen intensity over time. The taxa include *Ambrosia, Celtis*, Cupressaceae, *Morus/Broussonetia, Platanus*, Poaceae, *Quercus*, and *Ulmus*. Descriptive statistics of the pollen season metrics are shown in Table 3.

**Table 3:** Descriptive statistics of pollen seasons for the most abundant airborne pollen taxa in Tulsa, Oklahoma.

**Pollen Taxa**	**Years of Sampling**	**Mean Start Date DOY (Range)**	**Mean End Date DOY (Range)**	**Mean Duration Days (Range)**	**Mean Peak Date DOY (Range)**	**Mean Peak Concentration Pollen grains/m^**3**^ (Range)**	**Mean APIn/SPIn (Range)**
*Ambrosia*	34	233 (223–240)	300 (280–321)	68 (47–91)	254 (241–270)	847 (266–2,367)	11,217 (5,024–23,129)
*Celtis*	25	85 (72–101)	106 (92–135)	21 (5–45)	95 (78–110)	507 (49–2,179)	2,381 (316–5,428)
CupressaceaeSpring Cupressaceae	34	42 (32–69)	89 (69–123)	48 (11–92)	60 (41–75)	1,680 (442–4,214)	6,886 (1,708–17,451)
Fall Cupressaceae *Juniperus pinchotii*	34	N.A.	N.A.	N.A.	296 (268–318)	121 (10–1,051)	407 (54–1,924)
Winter Cupressaceae *Juniperus ashei*	34	N.A.	N.A.	N.A.	11 (356–30)	888 (97–3,513)	1,834 (326–6,098)
*Morus/Broussonetia*	34	87 (63–109)	125 (104–141)	39 (15–65)	100 (77–127)	750 (125–1,698)	4,739 (674–8,266)
*Platanus*	25	87 (72–107)	118 (106–132)	32 (16–44)	100 (85–119)	825 (275–1,613)	4,838 (1,875–8,818)
Poaceae	33	105 (74–125)	289 (265–309)	185 (161–215)	143 (134–161)	130 (57–263)	3,046 (1,772–5,053)
*Quercus*	33	79 (61–100)	121 (103–138)	43 (23–67)	99 (78–113)	1,899 (486–4,734)	14,093 (4,685–27,211)
*Ulmus:* Spring *Ulmus*	34	40 (25–70)	79 (62–102)	40 (20–68)	53 (34–71)	638 (150–1,444)	3,803 (934–6,480)
Fall *Ulmus*	25	239 (201–263)	277 (261–314)	39 (12–101)	260 (226–277)	333 (4–1,280)	1,034 (34–3,244)

*Since Cupressaceae and Ulmus have multiple species with different pollen seasons, the season pollen integral (SPIn) was determined for these taxa. The annual pollen integral (APIn) is shown for all other taxa. DOY is day of year starting with 1 January. N.A., not applicable, since these taxa are not local, the season start, end, and duration were not determined*.

#### Ambrosia Pollen

**Figure 4:**
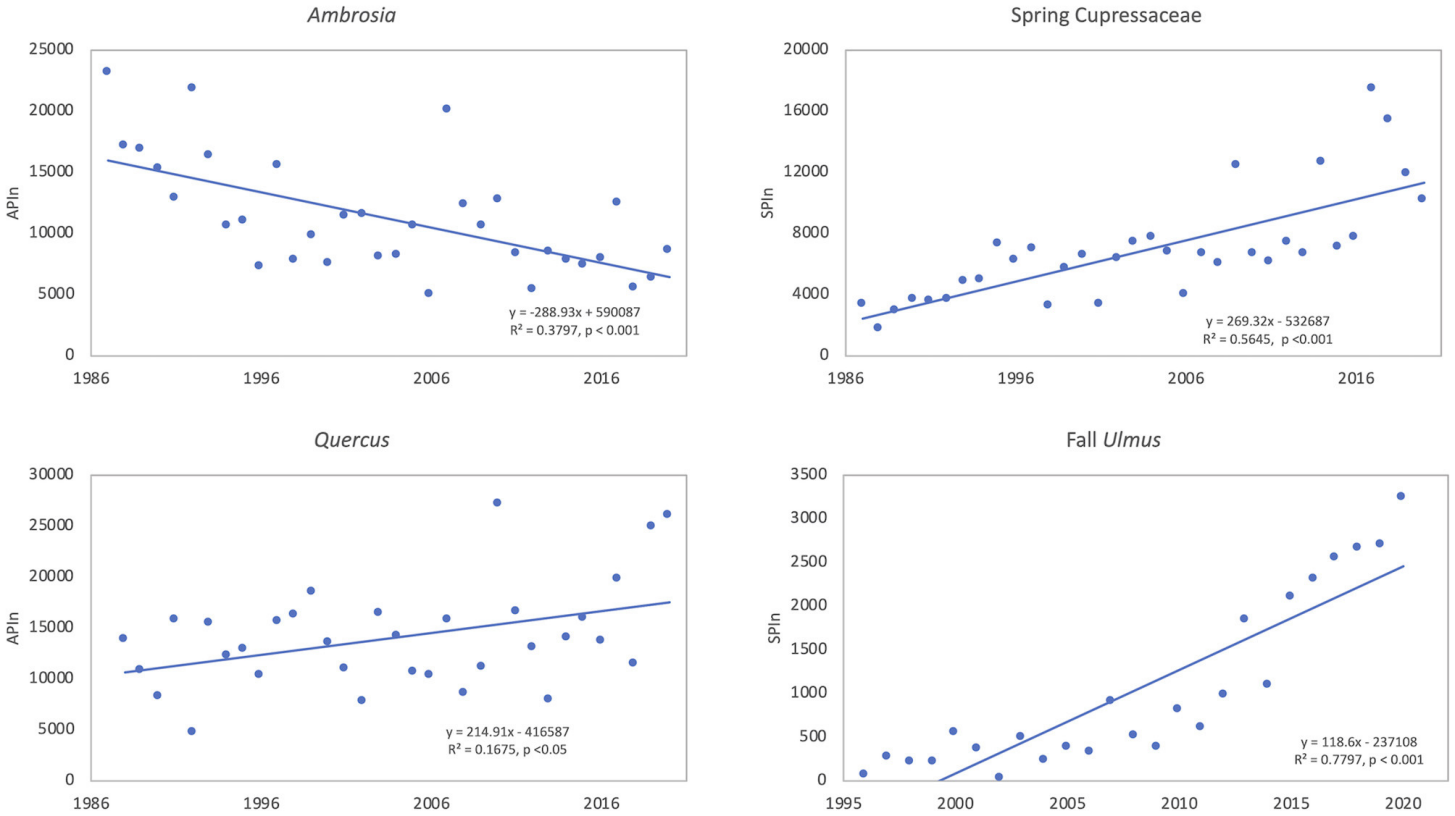
Linear trends of pollen intensity for taxa showing significant (*p* < 0.05) trends over time. The annual pollen integral (APIn) is shown for *Ambrosia* (34 years, 1987–2020) and *Quercus* (33 years, 1988–2020). The season pollen integral (SPIn) is shown for spring Cupressaceae (34 years, 1987–2020) and fall *Ulmus* (25 years, 1996–2020) since Cupressaceae and *Ulmus* have multiple species with different pollen seasons.

**Figure 5:**
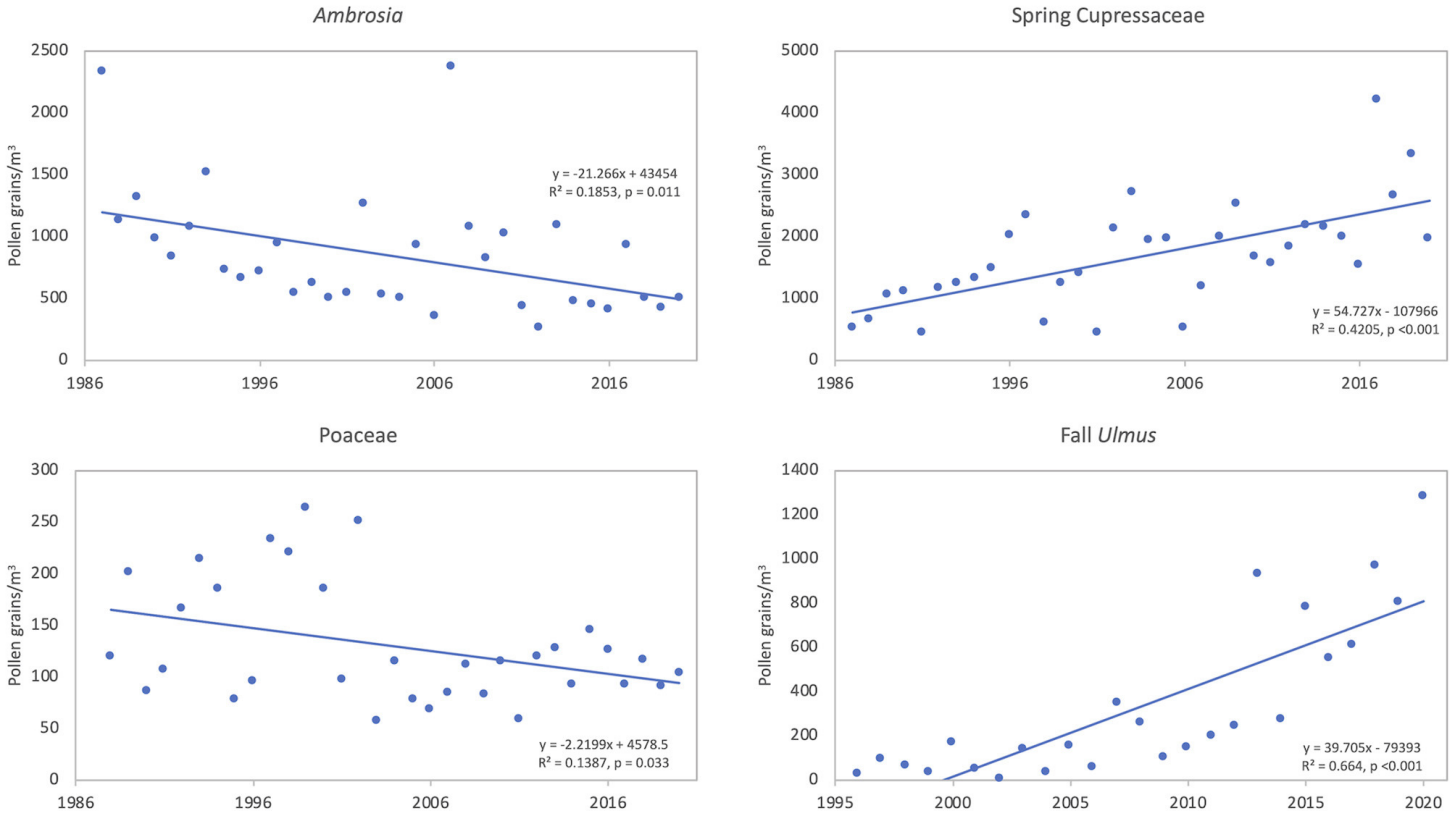
Linear trends of peak concentration for *Ambrosia* (34 years, 1987–2020), spring Cupressaceae (34 years, 1987–2020), Poaceae (33 years, 1988–2020), and fall *Ulmus* (25 years, 1996–2020). These taxa show significant (*p* < 0.05) trends over time.

**Figure 6:**
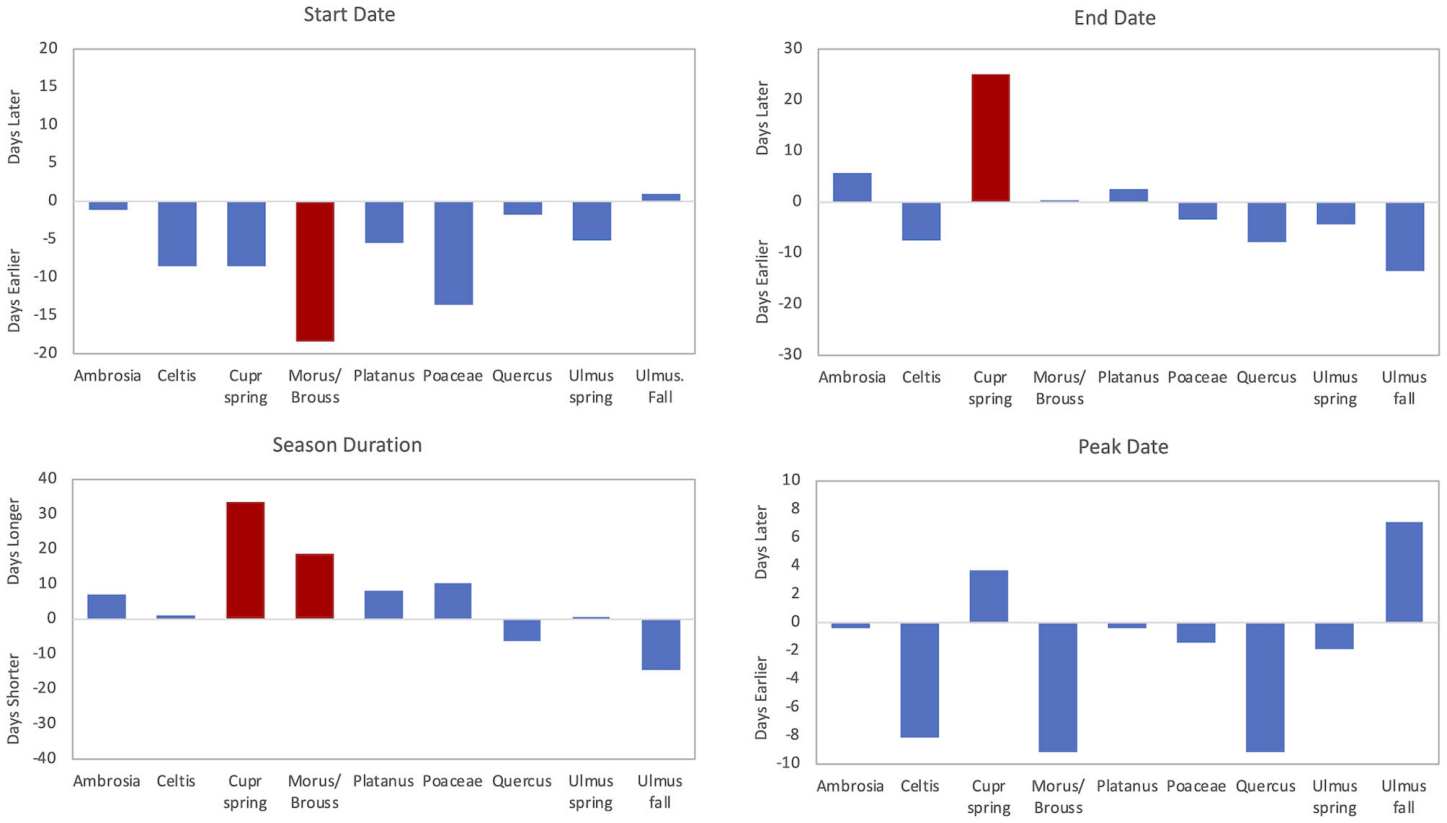
Trends for pollen season start date, end date, duration, and peak date over time. Red bars are significant (p < 0.05) trends, and blue bars show non-significant trends. *Ambrosia*, spring Cupr (Cupressaceae), *Morus/Broussonetia*, and spring *Ulmus* reflect trends over 34 years (1987–2020). Poaceae and *Quercus* reflect trends over 33 years (1988–2020). *Celtis, Platanus*, and fall *Ulmus* reflect trends over 25 years (1996–2020).

*Ambrosia* pollen had a mean APIn for the period at 11,217 (Table 3). The lowest APIn was 5,024 registered in 2006, and the highest was 23,129 in 1987. The mean season start and end dates were 21 Aug (day of year, DOY 233) and 27 October (DOY 300), respectively. Linear regression analysis showed declines in pollen intensity from 1987 to 2020 (Table 4, Figures 4, 5). There were significant decreases in both the APIn (R^2^ = 0.380, *p* < 0.001) and peak concentration (R^2^ = 0.185, *p* = 0.011) with a 62.7% decrease in APIn and 78.3% decrease in peak concentration. Linear regression of pollen season timing variables produced no significant results (Table 5), although there were trends (Figure 6) toward a later end date and a longer season duration.

**Table 4:** Temporal changes in pollen intensity for the major airborne pollen taxa in Tulsa, Oklahoma.

**Pollen Taxa**	**APIn or SPIn**	**Peak Concentration**
*Ambrosia*	**0.380 (−288.929)** ***p** **<*** **0.001**	**0.180 (−21.266)** ***p** **<*** **0.05**
*Celtis*	0.009 (−21.444) *p =* 0.660	0.010 (6.968) *p =* 0.634
Cupressaceae		
Spring Cupressaceae	**0.565 (269.315)** ***p** **<*** **0.001**	**0.420 (54.727)** ***p** **<*** **0.001**
Fall Cupressaceae *Juniperus pinchotii*	0.021 (6.068) *p =* 0.413	0.011 (2.129) *p =* 0.559
Winter Cupressaceae*Juniperus ashei*	0.230 (36.481) *p =* 0.191	0.030 (14.427) *p =* 0.329
*Morus/Broussonetia*	0.010 (19.032) *p =* 0.579	0.001 (1.378) *p =* 0.854
*Platanus*	0.011 (−28.932) *p =* 0.618	0.113 (−17) *p =* 0.100
Poaceae	0.011 (−8.091) *p =* 0.565	**0.139 (−2.220)** ***p** **=*** **0.033**
*Quercus*	**0.168 (214.910)** ***p** **=*** **0.018**	0.091 (32.263) *p =* 0.089
*Ulmus:*		
Spring *Ulmus*	0.030 (−23.886) *p =* 0.325	0.031 (−5.543) *p =* 0.322
Fall *Ulmus*	**0.780 (118.596)** ***p** **<*** **0.001**	**0.664 (39.705)** ***p** **<*** **0.001)**

*R^2^, (slope), and significance of linear regression analysis of annual pollen integral (APIn) or seasonal pollen integral (SPIn) and peak concentration are shown. Since Cupressaceae and Ulmus have multiple species with different pollen seasons, the SPIn was determined for these taxa. The APIn is shown for all other taxa. Significant results are in boldface type*.

**Table 5:** Temporal analysis of the main pollen season start date, end date, season duration, and peak date for the major airborne pollen taxa in Tulsa, Oklahoma.

**Pollen Taxa**	**Start Date**	**End Date**	**Duration**	**Peak Date**
*Ambrosia*	0.007 (−0.033) *p =* 0.637	0.036 (0.169) *p =* 0.281	0.048 (0.203) *p =* 0.210	0.0003 (−0.012) *p =* 0.914
*Celtis*	0.098 (−0.343) *p =* 0.127	0.063 (−0.305) *p =* 0.224	0.0009 (0.038) *p =* 0.888	0.110 (−0.327) *p =* 0.105
Cupressaceae (spring)	0.080 (−0.250) *p =* 0.104	**0.318 (0.738)** ***p** **<*** **0.001**	**0.314 (0.987)** ***p** **<*** **0.001**	0.015 (0.108) *p =* 0.489
*Morus/Broussonetia*	**0.268 (−0.542)** ***p** **=*** **0.002**	0.0001 (0.011) *p =* 0.948	**0.229 (0.553)** ***p** **=*** **0.004**	0.066 (−0.269) *p =* 0.143
*Platanus*	0.045 (−0.218) *p =* 0.306	0.017 (0.109) *p =* 0.538	0.130 (0.328) *p =* 0.076	0.0002 (−0.013) *p =* 0.953
Poaceae	0.095 (−0.409) *p =* 0.082	0.011 (−0.101) *p =* 0.563	0.041 (0.308) *p =* 0.259	0.005 (−0.042) *p =* 0.686
*Quercus*	0.003 (−0.054) *p =* 0.747	0.092 (−0.236) *p =* 0.086	0.031 (−0.182) *p =* 0.329	0.094 (−0.276) *p =* 0.082
*Ulmus* (spring)	0.022 (−0.149) *p =* 0.398	0.015 (−0.129) *p =* 0.488	0.0002 (0.020) *p =* 0.929	0.003 (−0.05) *p =* 0.744
*Ulmus* (fall)	0.0003 (0.044) *p =* 0.925	0.090 (−0.539) *p =* 0.146	0.050 (−0.583) *p =* 0.282	0.038 (0.286) *p =* 0.350

*R^2^, (slope), and significance of linear regression analysis of pollen season timing variables are shown. Significant results are in boldface type*.

Pollen variables were correlated with maximum and minimum monthly temperatures. Pollen season start date had a significant negative correlation with March T-max and a significant positive correlation with August T-min (Table 6). Peak date had significant positive correlations with August T-max and T-min (Supplementary Table 3). There were no significant correlations between monthly temperatures and APIn; however, peak concentration had significant negative correlations with April and July T-max (Supplementary Tables 4, 5). Peak date had a significant negative correlation with April precipitation, and peak concentration had a significant positive correlation with September precipitation (Supplementary Table 6).

**Table 6:** Correlation of the pollen season start dates with mean monthly maximum and minimum temperatures.

		**Jan**	**Feb**	**Mar**	**Apr**	**May**	**Jun**	**Jul**	**Aug**	**Sep**	**Oct**
*Ambrosia*	T-max	ns	ns	−0.408^*^	ns	ns	ns	ns	ns		
	T-min	ns	ns	ns	ns	ns	ns	ns	0.530^**^		
*Celtis*	T-max	ns	ns	−0.737^***^	ns						
	T-min	ns	ns	−0.704^***^	ns						
Cupressaceae (spring)	T-max	ns	−0.474^**^	ns							
	T-min	ns	ns	ns							
*Morus/Broussonetia*	T-max	ns	ns	−0.654^***^							
	T-min	ns	ns	−0.545^***^							
*Platanus*	T-max	ns	ns	−0.627^***^							
	T-min	ns	ns	−0.716^***^							
Poaceae	T-max	ns	ns	ns	ns						
	T-min	ns	ns	ns	ns						
*Quercus*	T-max	ns	−0.423^*^	−0.601^***^							
	T-min	ns	−0.510^**^	−0.620^***^							
*Ulmus* (spring)	T-max	−0.345^*^	−0.511^**^								
	T-min	ns	−0.481^**^								
*Ulmus* (fall)	T-max	ns	ns	ns	ns	ns	ns	ns	0.463^*^		
	T-min	0.460^*^	ns	ns	ns	ns	ns	0.432^*^	ns		

*Statistical significance is indicated by*

* *p < 0.05*;

** *p < 0.01*;

*** *p < 0.001*;

*ns is non-significant. Blank spaces are months beyond the season start and are not included*.

#### Celtis Pollen

The mean *Celtis* APIn was 2,381 (Table 3); the lowest APIn was 316 in 1996 and the largest was 5,428 in 2007. The mean start date was 26 March (DOY 85) and the mean end date was 16 April (DOY 106). There were no significant results from linear regression analysis of APIn, peak concentration, or pollen season timing variables (Tables 4, 5). However, there were trends toward earlier start date, end date, and the peak date (Figure 6).

Start date, end date, and peak date had significant negative correlations with March T-max and T-min. For start date and end date, the higher correlation coefficients were with T-max (Tables 6, 7), while peak date (Supplementary Table 3) had a higher coefficient with T-min. APIn showed significant negative correlations with January T-max, and peak concentration had significant negative correlations with both January and February T-max (Supplementary Tables 4, 5). There were no significant correlations between any *Celtis* pollen season metrics and monthly precipitation.

**Table 7:** Correlation of the pollen season end dates with mean monthly maximum and minimum temperatures.

		**Jan**	**Feb**	**Mar**	**Apr**	**May**	**Jun**	**Jul**	**Aug**	**Sep**	**Oct**
*Ambrosia*	T-max	ns	ns	ns	ns	ns	ns	ns	ns	ns	ns
	T-min	ns	ns	ns	ns	ns	ns	ns	ns	ns	ns
*Celtis*	T-max	ns	ns	−0.464^*^	ns	ns					
	T-min	ns	ns	−0.461^*^	ns	ns					
Cupressaceae (spring)	T-max	ns	−0.358^*^	ns	ns	ns					
	T-min	ns	−0.348^*^	ns	−0.343^*^	ns					
*Morus/ Broussonetia*	T-max	ns	ns	−0.349^*^	−0.465^**^	ns					
	T-min	−0.412^*^	ns	−0.479^**^	−0.528^**^	ns					
*Platanus*	T-max	ns	ns	ns	−0.495^*^	ns					
	T-min	ns	ns	−0.414^*^	−0.573^**^	ns					
Poaceae	T-max	ns	ns	ns	ns	ns	ns	ns	ns	ns	ns
	T-min	ns	ns	ns	ns	ns	ns	ns	ns	ns	ns
*Quercus*	T-max	ns	ns	−0.548^***^	−0.555^***^	ns					
	T-min	ns	ns	−0.546^**^	−0.543^**^	ns					
*Ulmus* (spring)	T-max	ns	−0.450^**^	ns	ns						
	T-min	ns	−0.387^*^	−0.437^**^	ns						
*Ulmus* (fall)	T-max	ns	ns	ns	ns	ns	ns	ns	0.507^*^	ns	ns
	T-min	ns	ns	ns	ns	ns	ns	ns	0.505^*^	ns	ns

*Statistical significance is indicated by*

* *p < 0.05*;

** *p < 0.01*;

*** * p <0.001*;

*ns is non-significant. Blank spaces are months beyond the season end date and are not included*.

#### Cupressaceae Pollen

Cupressaceae pollen represents approximately 16% of registered pollen in the Tulsa atmosphere (Supplementary Table 1) and occurs in three distinct pollen seasons: spring, fall, and winter. Data for the three seasons are described separately.

During the study period, the mean SPIn for the spring Cupressaceae pollen season was 6,886 with the range in SPIn from 1,708 in 1988 to 17,451 in 2017 (Table 3). The mean pollen season start date was 11 Feb (DOY 42), and the mean end date was 30 March (DOY 89). Linear regression analysis of spring Cupressaceae pollen intensity (Table 4) showed significant increases over time (Figures 4, 5) in both SPIn (R^2^ = 0.564, *p* < 0.001) and peak concentration (R^2^ = 0.420, *p* < 0.001). There was a 204.8% increase in the SPIn and a 276.5% increase in the peak concentration over the 34 years. Analysis of pollen season timing variables (Table 5) showed significant increases in the season end date and duration. The end date was 25.1 days later and the duration was 33.6 days longer. There was a non-significant trend of an earlier start date.

The start, end, and peak dates of the spring Cupressaceae season were significantly associated with February temperatures. Start date (Table 6) was negatively associated with February T-max, and end date (Table 7) was negatively correlated with both T-max and T-min. April T-min was also negatively correlated with season end date although the main pollen season often ended in late March. Peak date (Supplementary Table 3) was also negatively associated with February temperatures; T-min had the higher correlation coefficient. There were no significant correlations with monthly temperatures and either the SPIn or peak concentration (Supplementary Tables 4, 5). Pollen season metrics had no significant correlations with monthly precipitation.

During the fall Cupressaceae season in Tulsa, the registration of pollen is sporadic occurring on an average of 33% of the days during this period. The average SPIn in Tulsa was 407 with the range from 54 in 2011 to 1,924 in 2007 (Table 3). Linear regression analysis over the 34 years (Table 4) showed there was no significant change in SPIn or peak concentration.

The winter Cupressaceae pollen season in Tulsa is also sporadic occurring on an average of 48% of the days in December and January. The average SPIn for winter Cupressaceae pollen in Tulsa was 1,834 with the lowest value 326 in 2001 and the highest value 6,098 in 2017 (Table 3). Linear regression analysis of pollen intensity (Table 4) showed there were no significant trends in SPIn or peak concentration.

#### Morus/Broussonetia Pollen

*Morus* and *Broussonetia* pollen were grouped together for microscopy and data analysis. The mean APIn was 4,739 (Table 3). The lowest APIn was 674, which was registered in 1987, and the highest was 8,266 in 2012. The mean pollen season start date was 28 March (DOY 87) and the mean end date was 5 May (DOY 125). There were no significant changes in pollen intensity over time (Table 4). Linear regression analysis of season timing variables (Table 5, Figure 6) showed a significantly earlier start date (18.4 days) and a significantly longer pollen season duration (18.8 days). Although not significant, there was also a trend toward an earlier peak date (Figure 6).

Correlation of pollen season timing variables with monthly temperature produced several significant results for start date, end date, and peak date. Pollen season start date (Table 6) had significant negative correlations with March T-max and T-min. End date had significant negative correlations with January T-min as well as March and April T-max and T-min (Table 7). Peak date (Supplementary Table 3) had significant negative correlations with March T-max and T-min, and April T-min. There were no significant correlations between monthly temperature and APIn, while concentration had significant negative correlations with January T-min and February T-Max and T-min (Supplementary Tables 4, 5). There were no significant correlations for any pollen season metrics with monthly precipitation.

#### Platanus Pollen

The mean *Platanus* APIn during this period was 4,838 with the range from 1875 registered in 2018 to 8,818 in 2006 (Table 3). The mean pollen season start date was 28 March (DOY 87) and the mean end date was 30 April (DOY 118). Regression analysis of pollen intensity and pollen season timing variables found no significant changes over time (Tables 4, 5). Although not significant, there were slight trends toward an earlier start date and longer season duration (Figure 6).

Correlation of start date with monthly temperatures showed significant negative correlations with March T-max and T-min (Table 6). March T-min, April T-max, and April-T-min had significant negative correlations with season end date (Table 7). There were also significant negative correlations for peak date with March T-max, March T-min, and April T-min (Supplementary Table 3). There were no significant correlations for APIn or peak concentration with monthly temperature variables (Supplementary Tables 4, 5) and no significant correlations for any *Platanus* pollen metrics with monthly precipitation.

#### Poaceae Pollen

Mean APIn for Poaceae was 3,046 (Table 3). The lowest APIn was 1,772 recorded in 1995 and the highest was 5,053 in 1998. Poaceae pollen has the longest season duration of the major pollen types in Tulsa with the mean season start date on 15 April (DOY 105) and the mean end date on 16 October (DOY 289). Linear regression of the APIn over time showed a trend to lower concentrations but no significant change; however, there was a significant decrease (R^2^ = 0.139, *p* = 0.033) in the peak concentration during the 33 years (Table 4, Figure 5). Regression analysis of the pollen season timing variables (Table 5) showed no significant changes. However, there were trends toward an earlier start date and longer season duration (Figure 6).

Monthly temperature variables had very few significant correlations with Poaceae pollen season metrics (Tables 6, 7, Supplementary Tables 3–5). Peak date had significant negative correlations with May T-max and T-min and significant positive correlations with January T-min. Peak date also had significant positive correlations with April precipitation, while end date had significant negative correlations with February precipitation and significant positive correlations with September precipitation (Supplementary Table 6).

#### Quercus Pollen

*Quercus* pollen is the most abundant pollen type in the Tulsa atmosphere representing ~22.5% of total airborne pollen (Supplementary Table 1). The mean APIn was 14,093 (Table 3), and the range was from 4,685 in 1992 to 27,211 in 2010. Mean start date was 20 March (DOY 79) and mean end date was 1 May (DOY 121). Regression analysis of the APIn showed a significant increase (R^2^= 0.168, *p* = 0.018) from 1988 to 2020 (Table 4, Figure 4). This represents an 88% increase in pollen intensity. The peak concentration also had an increasing trend (Table 4) but it was not significant at the 5% level. There was no significant changes in pollen season timing variables (Table 5); however, the data show some trends with earlier peak date and end date (Figure 6).

There were several significant correlations between pollen metrics and monthly temperatures. Start date (Table 6) had significant negative correlations with February and March T-max and T-min. End date (Table 7) had significant negative correlations with March and April T-max and T-min. Peak date (Supplementary Table 3) had significant negative correlations with March T-Max and T-min. There was a significant negative correlation between APIn (Supplementary Table 4) and January T-max, and peak concentration (Supplementary Table 5) had significant negative correlations with January T-max and T-min. Pollen season metrics had no significant correlations with monthly precipitation.

#### Ulmus Pollen

*Ulmus* pollen occurs in two distinct seasons, spring and fall. There were 34 years of data for the spring *Ulmus* season and 25 years for the fall season. Data for the two seasons were analyzed separately.

The spring *Ulmus* season had a mean APIn of 3,803 (Table 3); the lowest APIn was 934 which was registered in 1989 and the highest was 6,480 in 1997. The mean start date was 9 February (DOY 40) and the mean end date was 20 March (DOY 79). Linear regression analysis of all pollen season metrics showed no significant changes over time (Tables 4, 5). Although not significant, the data showed slight trends toward an earlier pollen season (Figure 6).

Several spring *Ulmus* pollen metrics had significant associations with monthly temperatures. Start date (Table 6) had significant negative correlations with January T-max, February T-max, and February T-min. End date (Table 7) had significant negative correlations with February T-max, February T-min, and March T-min. Peak date (Supplementary Table 3) also had significant negative correlations with February T-max and T-min. There were no significant correlations for SPIn or peak concentration with monthly temperature variables (Supplementary Tables 4, 5), and no significant correlations for any spring *Ulmus* pollen season metrics with monthly precipitation.

The fall *Ulmus* season had a mean APIn of 1,034 with the range from 34 in 2002 to 3,244 in 2020 (Table 3). The mean start date was 27 August (DOY 239) and the mean end date was 4 October (DOY 277). Linear regression showed significant increases in SPIn (R^2^ = 0.780, *p* < 0.001) and peak concentration (R^2^ = 0.664, *p* < 0.001) during the 25 years (Table 4, Figures 4, 5). There were no significant changes in the pollen season timing variables (Table 5); however, there were trends in season timing with an earlier end date, shorter season duration, and later peak date (Figure 6).

Correlations with monthly temperature and pollen season timing variables showed several significant relationships (Tables 6, 7, Supplementary Table 3). Start date was positively correlated with January T-min, July T-min and August T-max. End date and peak date were positively correlated with August T-max and T-min. SPIn had significant positive correlations (Supplementary Table 4) with Mar T-max, Mar-T-min, Jun T-min, and September T-min. Peak concentration (Supplementary Table 5) was positively correlated with March T-max, Jun T-min and September T-min. Start date was positively correlated with January precipitation, and peak date was negatively correlated with August precipitation.

## Discussion

Yearly temperatures in Tulsa, Oklahoma have exhibited trends toward increasing T-max, T-min, and T-mean, with a significant increase in T-max (Table 1; Figure 2) from 1987 to 2020. Recent studies have shown that the increasing temperatures have resulted in changes in allergenic pollen season timing as well as pollen intensity (10, 11, 13, 15). The 25 to 34 years of airborne pollen data presented here also reflect changes in both pollen season timing and intensity. Precipitation in Tulsa was highly variable but showed no significant changes over time (Table 2). Only a few long-term pollen studies analyzed changes in precipitation over time. Gehrig and Clot (71) reported no significant changes in monthly or annual precipitation in Basel, Switzerland over 50 years. In Thessaloniki, Greece Damialis et al. (72) found no significant changes in annual precipitation over 19 years, and Velasco-Jiménez et al. (14) showed there were no significant changes in precipitation during the September to March period in three cities in southern Spain over 24 years. Although the climates in these three regions are different than Tulsa, there were similar results in the trend analyses.

### Total Airborne Pollen

Trends in APIn of total airborne pollen, tree pollen, weed pollen, and Poaceae pollen were analyzed from 1996 to 2020. The data for total pollen and tree pollen displayed increasing trends, while weed pollen and Poaceae pollen decreased (Figure 3). The increase in tree pollen was significant and likely reflects the significant increases in pollen intensity for spring Cupressaceae pollen, fall *Ulmus* pollen and *Quercus* pollen. These trends parallel results from other studies. Ziska et al. (13) found significant increases in APIn in 12 out of 17 locations in the Northern Hemisphere with long-term airborne pollen records. Anderegg et al. (11) examined data from 60 sampling stations in North America and found an increase of 20.9% in APIn of total pollen from the aggregate of all station. Tree pollen showed the largest increase in APIn.

### Individual Pollen Taxa

The eight most abundant taxa were selected for detailed analysis in pollen intensity metrics and variables that describe pollen season timing. These taxa represent over 83% of the total airborne pollen registered in Tulsa during this study and represent important aeroallergens in the region.

#### Ambrosia Pollen

The intensity of *Ambrosia* pollen in Tulsa exhibited significant decrease over the 34 year study period (Table 4, Figures 4, 5). This decrease, which had been previously documented for 27 years (49), continued through 2020. There was a 62.7% decrease in APIn and a 78.3% decrease in peak concentration. It is possible that urban development may partly explain the decrease in pollen intensity; however, other studies have also reported reductions in pollen intensity for *Ambrosia*. Zhang et al. (10) included *Ambrosia* pollen as one of the five pollen types investigated at 50 NAB stations across the United States. They found a non-significant decrease of 3.1% in *Ambrosia* APIn at 20 of the NAB stations when comparing the data from 1994 to 2000 to data from 2001 to 2010. During these two periods, the peak concentration increased by 12.4%; however, this change was not significant. Although another Tulsa pollen monitoring station was included in the Zhang et al. study, the *Ambrosia* data from that station was not used in the analysis based on the Supporting Information. Anderegg et al. (11) reported a non-significant decrease in fall weed pollen integrals, while the APIn for weed pollen showed a significant increase. While no individual weed pollen taxa were identified, *Ambrosia* pollen is typically an important component of the fall weed pollen total. Damialis et al. (72) reported a 27% decrease in the APIn for *Ambrosia* over the period of 1996–2005 when compared to the APIn from 1987 to 1995 in Thessaloniki, Greece, and Glick et al. (9) reported no substantial change in *Ambrosia* pollen intensity over 31 years at 14 monitoring sites in Switzerland. By contrast, Ziello et al. (73) found increases in *Ambrosia* APIn at 44 sites with 5 of these sites (11%) showing significant increases. These findings were part of a large study of APIn from 97 locations in 13 European countries. The study spanned the period from 1977 to 2009, and the data provided from stations ranged from 10 to 28 years. The study presented results for 23 pollen taxa; however, not every station reported on every pollen type. In another study across Europe, Sikoparija et al. (74) analyzed *Ambrosia* pollen data from 2004 to 2013 at 242 monitoring sites and determined trends over time for pollen intensity from 143 sites, which had at least 8 years of data. Significant trends were found at 11 sites (8%) with seven showing significant decreases. The authors suggest that the locations with significant decreases may be related to the introduction of control measures or the presence of beetles that feed on *A. artemisiifolia*, while the significant increases represent areas where *Ambrosia* is expanding its range.

Although *Ambrosia* pollen intensity decreased in Tulsa, there were non-significant trends toward a later end date (5.8 days later) and a longer season duration (6.9 days longer) (Figure 6). This supports findings of previous studies (15–17) showing a longer *Ambrosia* pollen season. Ziska et al. (15) reported longer season from 1995 to 2009 in the central area of North America due to delay of first frost. However, this is not the explanation for Tulsa since there was no significant change in the date of the first frost in Tulsa (R^2^ = 0.029, *p* = 0.344) during the study period (data not shown).

Pollen metrics had significant correlations with temperature and precipitation. Pollen season start date had negative correlations with March T-max and positive correlations with August T-min (Table 6). Peak date also had significant positive correlations with August T-max and T-min and negative correlations with April precipitation (Supplementary Tables 3, 6). The positive correlations with August temperatures suggest that warmer August temperatures result in later *Ambrosia* season start and peak dates. Warmer temperatures in March and increased April rainfall may promote earlier germination of *Ambrosia* seeds and seedling development. Peak concentration had positive correlations with September rainfall (Supplementary Table 6) suggesting that rainfall may aid the final stages of floral development and pollen release. Peak concentration had negative correlations with April and July T-max (Supplementary Table 5). Additional studies are needed to understand the influence of April temperatures on peak concentration; however, warmer July temperatures may reduce pollen intensity. This is supported by the fact that 2012 which was the hottest year during the study period had the lowest peak concentration. Both July T-max and T-min (Table 1) showed an increasing temperature trend over the 34 year period. Previous research in Tulsa using a Rotorod sampler found similar results during 1980, another year with record heat (75). Makra et al. (76) reported similar findings in Szeged, Hungary and suggested that heat stress during hot summers impedes the ability to pollinate in *Ambrosia*. Further research is needed to determine if increasing temperature is the main driver for the decrease in *Ambrosia* APIn in Tulsa and other areas or if urban development, which has occurred in Tulsa during the study period (77, 78), is also a contributing factor.

#### Cupressaceae Pollen

Cupressaceae pollen was present during three pollen seasons. The spring Cupressaceae season which is primarily composed of *Juniperus virginiana* pollen constitutes 75.4% of total yearly Cupressaceae pollen registered in Tulsa. The fall Cupressaceae season, which consists of *J. pinchotii* pollen, is 4.5% of the total Cupressaceae pollen, and the winter Cupressaceae season is *J. ashei* pollen which constitutes 20.1%, of the total.

The fall and winter Cupressaceae pollen registered in Tulsa is from long distance transport of pollen from populations to the southwest. The closest populations of either species are ~220 km southwest of Tulsa and the main populations are over 400 km for *J. pinchotii* and over 700 km for *J. ashei*. There were no significant differences in the SPIn or peak concentration of either species registered in Tulsa from 1987 to 2020 (Table 4). Because the pollen sources are not local, the Tulsa data cannot determine pollen season timing events or the influence of temperature on pollen metrics. Long term pollen sampling data from the source areas are needed to resolve these issues.

The spring Cupressaceae pollen intensity significantly increased SPIn (by 204.8%) and peak concentration (by 276.5%) during the 34 years of air sampling (Table 4, Figures 4, 5). These results extend previous work that analyzed aspects of spring Cupressaceae pollen in Tulsa for 10 years (46) and 30 years (48). The increasing population of *J. virginiana* trees in Oklahoma and other states in the Great Plains region is well-documented (34, 36–40), and ecologists have offered many explanations for the increase including fire suppression, overgrazing by livestock, increasing CO_2_ levels, and climate. Possibly all these factors are involved in the changing Oklahoma landscape.

Atmospheric CO_2_ concentration in Oklahoma has increased during the study period based on data from the NOAA Global Monitoring Laboratory at the Southern Great Plains site in Lamont, Oklahoma (79). This monitoring site is ~155 km from the University of Tulsa air sampling station. Average yearly CO_2_ levels from this site have increased from 375 ppm in 2002 to 417 ppm in 2020 (79). This increase may have contributed to the encroachment and growth of *J. virginiana* trees within Oklahoma. In addition, the increased CO_2_ may also have contributed to the increased pollen intensity. Although there have been no studies showing that CO_2_ directly increases pollen production in *J. virginiana*, other species have shown increased pollen production under experimentally elevated CO_2_ (18–23). Clearly, more research is needed to determine the role of climate change and increasing CO_2_ concentrations in *J. virginiana* encroachment in the Oklahoma prairies and the increased pollen production in this species.

Increasing APIn has also been reported in other areas of the world. Damialis et al. (72) found that APIn of Cupressaceae pollen increased 89% between the two-sampling period in Thessaloniki, and Ziello et al. (73) found increases in Cupressaceae APIn at 41 sites (out of 53 reporting) with 10 sites showing significant increases in pollen intensity over time. The use of Cupressaceae trees in landscaping was mentioned as a partial explanation for the increase. Two studies from Spain (80, 81) covering 20 and 23 years showed there was a general increase in APIn of Cupressaceae pollen with a significant increase in several locations. Both studies found that the increase was especially prominent at sites where Cupressaceae trees were planted as ornamentals.

The data from the current study show signification increases in the season end date and duration; with the end date 25.1 days later and season duration 33.6 days longer (Table 5, Figure 6). There was also a non-significant trend of an earlier start date. Similar results in pollen season timing were found in Jaen, Spain (81) in a 23 year study. In the present study, there were significant negative correlations of start, end and peak date with February temperatures (Tables 6, 7, Supplementary Table 3) indicating that warmer February temperatures resulted in an earlier pollen season.

#### Poaceae Pollen

For Tulsa Poaceae pollen there was a trend to decreasing APIn over the 33 years; however, the decrease was not significant (Table 4). Peak concentration did show a significant decrease during this time (Table 4, Figure 5). Results from other locations were inconsistent with some locations showing increases in APIn and others showing decreases. Zhang et al. (10) found a 43.4% increase in APIn and a 23% increase in peak concentration from 26 NAB stations when comparing data from the two time periods, but neither increase was statistically significant. In their analysis of Poaceae data from NAB sites, Anderegg et al. (11) showed a small decrease in the summer Poaceae pollen integral, but an increase in the annual integral for Poaceae pollen. Neither of these trends was significant. Ziello et al. (73) found that there was no significant change in the composite Poaceae data of all the study sites in Europe, but 29 individual sites (out of 82) had trends toward increased Poaceae APIn and 8 of these sites had significant increases. Non-significant increasing trends were also found in Switzerland (9) and Jaen, Spain (81). In an analysis of 20 years of pollen data from 12 cities in Spain, Galán et al. (80) found increasing trends in Poaceae pollen intensity at most sites and significant increases at four sites. In Greece Damialis et al. (72) determined there was a 199% increase in Poaceae pollen intensity between the two sampling periods.

By contrast, Hoebeke et al. (82) reported a significant decrease in Poaceae APIn over 34 years in Brussels, and suggested that increased urbanization reduced available land for herbaceous vegetation. Land use changes has been suggested as a major factor in other studies as well. Emberlin et al. (83) analyzed long-term datasets of airborne Poaceae pollen from three regions in the United Kingdom. Yearly pollen intensity decreased over time in London and Derby but fluctuated greatly in Cardiff. The authors found that these trends were similar to changes in total area of grasslands surrounding each location. In addition, they also found that preseason temperature and precipitation also influenced pollen intensity at each site. Gehrig and Clot (71) found no change in the APIn for Poaceae over 50 years in Basel, Switzerland. The authors suggest that the increase in urbanization and decrease in agricultural land around Basel may have been responsible for the lack of change in Poaceae pollen as well as the general decrease in the APIn of herbaceous species. Increased urbanization and land use changes may also be a factor in the decreasing trend of Poaceae pollen intensity in the current study. The Tulsa metropolitan area (the city of Tulsa and surroundings suburban cities and towns) has seen significant growth since 1990, especially in the suburbs (77). During this time the population grew by 32% reaching a population over one million by July 2020 (78). Wilson and Wilson (77) showed that the increase in residential land resulted from the conversion of forest, agricultural, and grass or shrub land. These studies showing increases, no change, or decreases in Poaceae pollen intensity may indicate that there is no universal response to climate change among grasses. The varying responses may reflect different species of grasses, changes in land use, local climatic differences or a combination of factors.

Linear regression of the pollen season timing variables for Poaceae pollen found no significant trends. Although not significant, the start date was 13.5 days earlier and the season duration 10.2 days longer (Figure 6). Zhang et al. (10) reported a shorter season duration with a 0.2 day earlier season start and a 4.8 earlier end date across 26 NAB stations. For the 14 pollen monitoring stations in Switzerland, Glick et al. (9) reported a significantly earlier start date ranging from 8 to 25 days based on the criteria used for defining the main pollen season metrics. They also found a longer season with four of the six definitions showing a significantly longer pollen season. Hoebeke et al. (82) also reported a significantly earlier start date and end date.

There were few significant correlations between pollen season metrics and temperature (Supplementary Table 3). Peak date had a positive correlation with Jan T-min, implying that higher T-min in January resulted in later peak dates. This may suggest some interference with the chilling requirement for seed germination in native grasses. May T-max and T-min had negative correlations with peak date suggesting warmer May temperatures resulted in an earlier peak date; however, positive correlations with April rainfall, suggest a later peak date. The mean peak date was 23 May. February rainfall had negative correlations with end date and September rainfall had positive correlations with end date, possibly reflecting differing responses among different grass species.

#### Quercus Pollen

There are 26 species of *Quercus* native to Oklahoma. The Post Oak-Blackjack Oak (*Q. stellata* and *Q. marilandica*) plant community is found in parts of Tulsa and extends both west and south (49, 50). Along with other woody species, this community is often referred to as the Cross-Timbers due to the density of the vegetation. The Post Oak-Blackjack Oak community intergrades with the Tallgrass Prairie in central Oklahoma. Studies have shown that *Q. marilandica* is often a partner to *Juniperus virginiana* in woody plant encroachment into the prairie (39). The native vegetation plus the widespread use of *Quercus* in the urban landscape explain why *Quercus* pollen is the most abundant pollen type in the atmosphere.

Data from this study (Table 4, Figure 4) show significant increases in *Quercus* APIn representing an 88% increase in pollen intensity over 33 years. Although not significant, the peak concentrations also showed an increasing trend with a 106% increase. Landscaping changes on campus over the past 15 years may have contributed to some of this increase as oak trees were planted in several areas around the campus. Although the impact of campus plantings cannot be ignored, these data are similar to the results from NAB stations in the United States. Zhang et al. (10) reported significant increases in both APIn (92.5%) and peak concentration (86.4%) from 28 sites between the two periods of time in their study. Damialis et al. (72) found a 136% increase in APIn as well as a significant increase in peak concentration between the two sampling periods in Thessaloniki. Glick et al. (9) also found a significant increase in APIn across the 14 monitoring sites in Switzerland. At sites across Europe, Ziello et al. (73) reported that 49 sites (out of 73) had increases in APIn for *Quercus* pollen with 4 locations having significant increases. In Spain, Galan et al. (80) showed that most locations had increases in APIn, and only 3 sites had decreases.

Although there were no significant changes in the season timing variables over 33 years in Tulsa, there were some trends for the *Quercus* pollen season with the end date 7.8 days earlier, peak date 9.1 days earlier, and season duration 6 days shorter (Figure 6). Zhang et al. (10) found the start date was 4.4 days earlier and the duration 3.1 days shorter at 28 sites. Glick et al. (9) reported a significantly earlier start date ranging from 5 to 13 days depending on the criteria used for defining the main pollen season metrics and also found a longer season duration. For four of the six definitions the pollen season was significantly longer. Ruiz-Valenzuela and Aguilera (81) indicated that peak date and end date were significantly later, but there was no significant change to the start date.

Correlations with pollen season timing variables and monthly temperatures found the warmer spring temperatures promoted earlier start, peak, and end dates for the *Quercus* pollen season in Tulsa (Tables 6, 7, Supplementary Table 3). These correlations tie in with the trends in monthly temperature, which showed that both March T-max and T-min had increasing trends over time, with 2.33 and 1.59°C increases, respectively (Table 1). APIn and peak concentration had significant negative correlations (Supplementary Tables 4, 5) with January T-max suggesting warmer January T-max may hamper the chilling requirement needed to end dormancy.

#### Ulmus Pollen

There are seven native or naturalized *Ulmus* species in Oklahoma (62). Four of these (*U. americana, U. alata, U. pumila*, and *U. rubra*) are considered spring pollinators and the other three (*U. crassifolia, U. parvifolia*, and *U. serotina*) are referred to fall pollinators. These were considered separately in the current study.

The spring *Ulmus* pollen season showed no significant change over 34 years in either pollen intensity metrics or season timing variables. However, there were small trends toward an earlier pollen season with the start date 5 days earlier and the end date 4.4 days earlier (Figure 6). Pollen season metrics had significant correlations with temperature variables in January through March, especially February T-max. These indicate that warmer temperatures led to earlier start date, peak date, and end date (Tables 6, 7, Supplementary Table 3).

Ziello et al. (73) found that there was no significant change in the composite data across all sites, but there were increasing trends in APIn at 28 sites (out of 33) and 6 sites showed significant increases. Ruiz-Valenzuela and Aguilera (81) noted an increase in *Ulmus* APIn that was significant at 10% level. There were no significant changes, but there were trends showing an earlier start date, later end date, and later peak date. In a 24 year analysis of winter pollinating trees from three cities of southern Spain, Velasco-Jiménez et al. (14) reported a significant increase in *Ulmus* APIn in Cordoba, a significant decrease in Granada, and no change in Malaga. The start date in Granada was significantly earlier. The authors also found that warmer temperatures months before pollination increased APIn in spring flowering *Ulmus* species, and indicated that the warmer temperatures promote the development of flower buds and consequently more pollen release. However, they also suggest that the warmer temperatures may impede the chilling requirement needed to end dormancy.

The fall *Ulmus* season had significant increases in both SPIn and peak concentration from 1996 to 2020 (Table 4, Figures 4, 5). Although regression analysis showed no significant changes over time for pollen season timing variables, there were trends in season timing. The end date was 13.5 days earlier, season duration was 14.6 days shorter, and peak date was 7.2 days later (Figure 6). There were several significant correlations between preseason temperatures and precipitation with pollen metrics (Tables 6, 7, Supplementary Tables 3–6). These correlations indicated that warmer temperatures these months result in later pollen start, end, and peak dates and increased pollen intensity. The January temperature correlation may suggest delays in the chilling requirement prior to leaf-out and spring development as suggested by Velasco-Jiménez et al. (14). Increased precipitation in January also resulted in a later start date, and more work is needed to understand this relationship. August precipitation had a negative relationship with peak date, suggesting that rain may accelerate final flower development and pollen release.

Most *Ulmus* species are affected by Dutch elm disease although disease severity varies with the species, and *Ulmus americana* is especially susceptible to the pathogen. The *U. americana* trees on campus fell victim to the disease more than 30 years ago, and trees in the city, state, and areas beyond continue to fall victim to the disease (84). Resistant cultivars of *U. americana* and other *Ulmus* species that are less susceptible are used in landscaping today; in Oklahoma these include *U. crassifolia*, which is only moderately susceptible to the disease, and *U. parvifolia*, which is resistant. Both are fall pollinating species (62, 85) and *U. parvifolia* is widely recommended for use. This is an introduced species that has become naturalized in Oklahoma and many other states. It spreads easily and is considered an invasive species in several states (86). The significant increase in fall *Ulmus* pollen intensity may be attributed to the use of *U. parvifolia* and *U. crassifolia* in landscaping as well as the natural spread of both species in the environment.

#### Celtis, Morus/Broussonetia, and Platanus Pollen

There were no significant changes in pollen season intensity for *Celtis, Morus/Broussonetia*, or *Platanus*. *Morus/Broussonetia* pollen had a significantly earlier start date of 18.4 days and a significantly longer pollen season of 18.8 days (Table 6, Figure 6). There was also a non-significant trend toward an earlier peak date. *Platanus* and *Celtis* had non-significant trends to earlier start dates, and *Celtis* had trends to an earlier end date and peak date (Figure 6). The three pollen taxa also had significant negative correlations between start date, end date and peak date with monthly temperatures (Tables 6, 7, Supplementary Table 3). Only March temperatures were significant for *Celtis*, while March and April temperatures had significant correlations for *Platanus* and *Morus/Broussonetia* season timing variables. These negative correlations between temperatures and pollen season timing variables suggest that warmer preseason temperatures were driving the trend toward earlier pollen seasons. The results of these correlations similar to those with *Quercus*, tie in with the trends of warmer T-max and T-min temperatures in March (Table 1). There was a significant negative correlation between January temperatures and *Celtis* APIn. Peak concentrations for *Celtis* and *Morus/Broussonetia* pollen also had significant negative correlations with January and February temperatures, possibly suggesting some interference with the chilling requirement for these taxa. However, more work needs to be done to understand these relationships since warmer January temperatures appear to promoted an earlier pollen season end date for *Morus/Broussonetia*.

In other areas, Makra et al. (87) showed that both *Morus* and *Platanus* had significant increases in pollen intensity over a 10 year period in Szeged, Hungary. García-Mozo et al. (88) also reported a significant increase in pollen intensity for both *Morus* and *Platanus* in Cordoba, Spain over a 15 year period. The authors attributed the change in *Morus* pollen intensity to increased urbanization in Cordoba. In Jaen, Spain (81) Ruiz-Valenzuela and Aguilera found a significant increase in *Platanus* pollen APIn over 23 years. There were no significant changes in start date or peak date, but the end date was significantly later and the pollen season significantly longer. In another study in Spain from multiple locations, Galan et al. (80) found no clear trend in changes over time for *Platanus* pollen. Some sites had increasing trends in APIn, others had decreasing trends, and very few trends were significant. Hoebeke et al. (82) reported significant increases in *Platanus* APIn and peak concentration. They also found a significantly earlier end to the *Platanus* pollen season. In Thessaloniki, Damialis et al. (72) showed a 522% increase in *Platanus* APIn between the two sampling periods. At sites across Europe Ziello et al. (73) found 42 sites (out of 52) showed increasing trends in APIn with 10 of these sites having significant increases in *Platanus* APIn. In general, these studies found increasing pollen intensity for *Morus* and *Platanus*; however, the Tulsa data for these taxa showed no significant change in pollen intensity but indications of an earlier pollen season for *Morus/Broussonetia*, as well as for *Platanus* and *Celtis*. More work is needed on these and other taxa to see if earlier pollen seasons or increased pollen intensity is occurring in other areas.

One of the limitations of this study was only counting certain pollen types during the first 9 years of sampling. The pollen types analyzed during those years were important aeroallergens in Tulsa based on discussions with local allergists. Another limitation was using different counting methods to analyze the Burkard slides over the 34 years; this introduces some uncertainty. However, when the single longitudinal traverse method was used, the slides were often recounted for specific pollen types, adding two or three additional traverses. As described above, there were highly significant correlations between the concentrations determined by these different counting methods. This suggests that any uncertainty from the use of multiple methods had minimal impact on the results, and the data accurately reflect the trends in airborne pollen in Tulsa.

## Conclusions

The current study addressed airborne allergenic pollen in the Tulsa, Oklahoma atmosphere over a period of 25 to 34 years. During this time yearly temperatures increased with a significant increase in T-max. The total pollen levels increased over time with significant increases in tree pollen. Both weed pollen and Poaceae pollen decreased, but not significantly. Detailed analyses focused on the eight most abundant airborne pollen taxa, which are also allergenic. This study found several significant changes over time with some taxa such as spring Cupressaceae and *Quercus* showing significant increases in pollen intensity and others like *Ambrosia* showing significant decreases. These different responses among species parallel results of long-term studies from other areas and indicates that plant taxa are not responding in a uniform manner to climate change. Landscape changes in both the natural environment and urban environment may partially explain some of the changes in pollen intensity. Temporal changes in pollen season timing were also analyzed with most taxa showing an earlier pollen season start. Data also show some statistically significant correlations with preseason temperature promoting an earlier pollen season for some taxa, which may be related to the trend toward increasing temperatures in March. Additional research is needed in other areas to understand the global impact of climate change on allergenic pollen, and consequently the impact on patients with pollen allergies.

## Data Availability Statement

The raw data supporting the conclusions of this article will be made available by the author, without undue reservation.

## Author Contributions

EL developed the concept of this study, analyzed the data, and wrote the manuscript.

## Conflict of Interest

The author declares that the research was conducted in the absence of any commercial or financial relationships that could be construed as a potential conflict of interest.

## Publisher's Note

All claims expressed in this article are solely those of the authors and do not necessarily represent those of their affiliated organizations, or those of the publisher, the editors and the reviewers. Any product that may be evaluated in this article, or claim that may be made by its manufacturer, is not guaranteed or endorsed by the publisher.
