# Supplementary material for: Aeroallergens and Climate Change in Tulsa, Oklahoma: Long-Term Trends in the South Central United States
Source: Front Allergy. 2021 Oct 7;2:726445. doi: 10.3389/falgy.2021.726445 (PMC8974782; doi:10.3389/falgy.2021.726445)
Supplement: Supplementary file 1 [file Data_Sheet_1.docx]

**Supplementary Material**

**Supplementary Table 1**. Mean APIn of airborne pollen in Tulsa, Oklahoma from 1996-2020. Other pollen includes Apiaceae, Brassicaceae, *Cannabis/Humulus*, *Corylus, Fagus, Ginkgo*, *Sambucus, Sapindus*, and *Tilia* pollen along with unknown and indeterminant pollen grains.

| Taxa | Mean APIn | Standard Deviation | Range | Percent of total pollen |
| --- | --- | --- | --- | --- |
| *Quercus* | 14,844 | + 5,274 | 7,824-27,211 | 22.56% |
| Cupressaceae | 10,443 | + 4,628 | 3,907-23,809 | 15.87% |
| *Ambrosia* | 9,465 | + 3,400 | 5,024-2,0089 | 14.39% |
| *Platanus* | 4,838 | + 2,029 | 1,875-8,818 | 7.35% |
| *Morus/Broussonetia* | 4,824 | + 1,516 | 1,826-8,265 | 7.33% |
| *Ulmus* | 4,757 | + 1,665 | 1,490-8,228 | 7.23% |
| Poaceae | 3,125 | + 690 | 2,234-5,053 | 4.72% |
| *Celtis* | 2,381 | + 1,704 | 316-5,428 | 3.62% |
| Pinaceae | 1,180 | + 621 | 349-3,044 | 1.79% |
| *Fraxinus* | 1,168 | + 1,284 | 93-4,946 | 1.78% |
| *Carya* | 1,166 | + 309 | 666-1,953 | 1.77% |
| *Betula* | 796 | + 445 | 211-1,794 | 1.21% |
| *Maclura* | 785 | + 391 | 192-1,566 | 1.19% |
| Urticaceae | 652 | + 718 | 66-3,008 | 0.99% |
| *Populus* | 624 | + 204 | 305-1,192 | 0.95% |
| Amaranthaceae | 563 | + 145 | 249-793 | 0.86% |
| *Salix* | 479 | + 217 | 64-1,141 | 0.73% |
| *Liquidamber* | 284 | + 127 | 39-520 | 0.43% |
| *Rumex* | 276 | + 128 | 82-638 | 0.42% |
| *Juglans* | 266 | + 104 | 80-493 | 0.40% |
| *Acer* | 253 | + 154 | 51-615 | 0.38% |
| Other Asteraceae | 191 | + 145 | 25-581 | 0.29% |
| *Artemisia* | 185 | + 117 | 17-564 | 0.28% |
| Cyperaceae | 158 | + 81 | 40-383 | 0.24% |
| *Plantago* | 121 | + 136 | 0-724 | 0.18% |
| *Xanthium* | 39 | + 55 | 0-194 | 0.06% |
| *Alnus* | 30 | + 26 | 0-91 | 0.04% |
| *Carpinus* | 28 | + 48 | 0-234 | 0.04% |
| *Ligustrum* | 24 | + 27 | 0-97 | 0.04% |
| *Typha* | 21 | + 28 | 0-105 | 0.03% |
| *Myrica* | 19 | + 19 | 0-71 | 0.03% |
| Juncaceae | 7 | + 8 | 0-28 | 0.01% |
| Other Pollen | 1,943 | + 673 | 440-3,067 | 2.95% |
| Total Pollen | 65,815 | + 11,877 | 46,282-86,902 | 100% |

**Supplementary Table 2**. Mean, maximum, and minimum yearly temperatures and total yearly precipitation in Tulsa, Oklahoma from 1987 to 2020. All temperature values are the average of the daily mean, maximum, or minimum temperature for each year. Meteorological data are from the Tulsa National Weather Service station approximately 8 km from the air sampling site.

| Year | T- Mean °C | T-Max °C | T-Min °C | Total Yearly Precipitation mm |
| --- | --- | --- | --- | --- |
| 1987 | 16.4 | 22.0 | 10.9 | 1166 |
| 1988 | 15.9 | 21.8 | 9.9 | 844 |
| 1989 | 15.1 | 20.9 | 9.4 | 892 |
| 1990 | 17.2 | 23.0 | 11.4 | 989 |
| 1991 | 17.0 | 22.6 | 11.4 | 852 |
| 1992 | 16.0 | 21.5 | 10.4 | 1102 |
| 1993 | 14.6 | 20.1 | 9.1 | 993 |
| 1994 | 15.7 | 21.4 | 10.0 | 1258 |
| 1995 | 15.7 | 21.5 | 9.9 | 1059 |
| 1996 | 15.3 | 21.3 | 9.3 | 855 |
| 1997 | 15.2 | 20.8 | 9.6 | 1048 |
| 1998 | 17.0 | 22.6 | 11.3 | 1180 |
| 1999 | 16.7 | 22.7 | 10.7 | 1231 |
| 2000 | 16.0 | 21.7 | 10.2 | 1044 |
| 2001 | 16.7 | 22.6 | 10.9 | 740 |
| 2002 | 16.0 | 21.8 | 10.2 | 793 |
| 2003 | 16.0 | 22.0 | 10.0 | 1049 |
| 2004 | 16.1 | 21.8 | 10.3 | 1257 |
| 2005 | 16.7 | 22.8 | 10.6 | 717 |
| 2006 | 17.4 | 23.8 | 11.0 | 971 |
| 2007 | 16.6 | 22.1 | 11.1 | 1348 |
| 2008 | 15.9 | 22.0 | 9.8 | 1425 |
| 2009 | 15.6 | 21.4 | 9.8 | 1171 |
| 2010 | 16.3 | 22.1 | 10.5 | 876 |
| 2011 | 17.1 | 23.4 | 10.8 | 813 |
| 2012 | 18.2 | 24.6 | 11.7 | 730 |
| 2013 | 15.3 | 21.3 | 9.3 | 840 |
| 2014 | 15.4 | 21.5 | 9.3 | 752 |
| 2015 | 16.6 | 22.5 | 10.7 | 1569 |
| 2016 | 17.7 | 23.8 | 11.6 | 716 |
| 2017 | 17.2 | 23.3 | 11.1 | 1169 |
| 2018 | 16.3 | 22.2 | 10.3 | 866 |
| 2019 | 16.1 | 21.8 | 10.4 | 1513 |
| 2020 | 16.5 | 22.4 | 10.6 | 1203 |

**Supplementary Table 3**. Correlation of the pollen season peak dates with mean monthly maximum and minimum temperatures. Statistical significance is indicated by *p < 0.05; ** p < 0.01; *** p < 0.001; ns is non-significant. Blank spaces are months beyond the season peak date and are not included.

|  |  | Jan | Feb | Mar | Apr | May | Jun | Jul | Aug | Sep | Oct |
| --- | --- | --- | --- | --- | --- | --- | --- | --- | --- | --- | --- |
| *Ambrosia* | T-max | ns | ns | ns | ns | ns | ns | ns | 0.378* | ns |  |
|  | T-min | ns | ns | ns | ns | ns | ns | ns | 0.514** | ns |  |
| *Celtis* | T-max | ns | ns | -0.794*** | ns |  |  |  |  |  |  |
|  | T-min | ns | ns | -0.809*** | ns |  |  |  |  |  |  |
| Cupressaceae (spring) | T-max | ns | -0.596*** | ns |  |  |  |  |  |  |  |
|  | T-min | ns | -0.634*** | ns |  |  |  |  |  |  |  |
| *Morus* | T-max | ns | ns | -0.546*** | ns |  |  |  |  |  |  |
|  | T-min | ns | ns | -0.532** | -0.482** |  |  |  |  |  |  |
| *Platanus* | T-max | ns | ns | -0.533** | ns |  |  |  |  |  |  |
|  | T-min | ns | ns | -0.630*** | -0.466* |  |  |  |  |  |  |
| Poaceae | T-max | ns | ns | ns | ns | -0.382* | ns |  |  |  |  |
|  | T-min | 0.379* | ns | ns | ns | -0.436* | ns |  |  |  |  |
| *Quercus* | T-max | ns | ns | -0.661*** | ns |  |  |  |  |  |  |
|  | T-min | ns | ns | -0.628*** | ns |  |  |  |  |  |  |
| *Ulmus* (spring) | T-max | ns | -0.474** | ns |  |  |  |  |  |  |  |
|  | T-min | ns | -0.443** | ns |  |  |  |  |  |  |  |
| *Ulmus* (fall) | T-max | ns | ns | ns | ns | ns | ns | ns | 0.399* | ns |  |
|  | T-min | ns | ns | ns | ns | ns | ns | ns | 0.425* | ns |  |

**Supplementary Table 4.** Correlation of the annual pollen integral (APIn) or seasonal pollen integral (SPIn) with the mean monthly maximum and minimum temperatures. Since Cupressaceae and *Ulmus* have multiple species with different pollen seasons, the SPIn was correlated with temperature for these taxa. The APIn was used in the correlation analysis for all other taxa. Statistical significance is indicated by *p < 0.05; ** p < 0.01; *** p < 0.001; ns is non-significant. Blank spaces are months beyond the season end and not included.

|  | | Jan | Feb | Mar | Apr | May | Jun | Jul | Aug | Sep | Oct |
| --- | --- | --- | --- | --- | --- | --- | --- | --- | --- | --- | --- |
| *Ambrosia* | T-max | ns | ns | ns | ns | ns | ns | ns | ns | ns | ns |
|  | T-min | ns | ns | ns | ns | ns | ns | ns | ns | ns | ns |
| *Celtis* | T-max | -0.429* | ns | ns | ns | ns |  |  |  |  |  |
|  | T-min | ns | ns | ns | ns | ns |  |  |  |  |  |
| Cupressaceae (spring) | T-max | ns | ns | ns | ns | ns |  |  |  |  |  |
|  | T-min | ns | ns | ns | ns | ns |  |  |  |  |  |
| *Morus* | T-max | ns | ns | ns | ns | ns |  |  |  |  |  |
|  | T-min | ns | ns | ns | ns | ns |  |  |  |  |  |
| *Platanus* | T-max | ns | ns | ns | ns | ns |  |  |  |  |  |
|  | T-min | ns | ns | ns | ns | ns |  |  |  |  |  |
| Poaceae | T-max | ns | ns | ns | ns | ns | ns | ns | ns | ns | ns |
|  | T-min | ns | ns | ns | ns | ns | ns | ns | ns | ns | ns |
| *Quercus* | T-max | -0.368* | ns | ns | ns | ns |  |  |  |  |  |
|  | T-min | ns | ns | ns | ns | ns |  |  |  |  |  |
| *Ulmus* (spring) | T-max | ns | ns | ns | ns |  |  |  |  |  |  |
|  | T-min | ns | ns | ns | ns |  |  |  |  |  |  |
| *Ulmus* (fall) | T-max | ns | ns | 0.424* | ns | ns | ns | ns | ns | ns | ns |
|  | T-min | ns | ns | 0.402* | ns | ns | 0.443* | ns | ns | 0.449* | ns |

**Supplementary Table 5**. Spearman correlation of peak concentrations with mean monthly maximum and minimum temperatures. Statistical significance is indicated by *p <0.05; ** p <0.01; ***p <0.001; ns is non-significant. Blank spaces are months beyond the season peak and not included.

|  |  | Jan | Feb | Mar | Apr | May | Jun | Jul | Aug | Sep | Oct |
| --- | --- | --- | --- | --- | --- | --- | --- | --- | --- | --- | --- |
| *Ambrosia* | T-max | ns | ns | ns | -0.358* | ns | ns | -0.392* | ns | ns |  |
|  | T-min | ns | ns | ns | ns | ns | ns | ns | ns | ns |  |
| *Celtis* | T-max | -0.442* | -0.459 | ns | ns |  |  |  |  |  |  |
|  | T-min | ns | ns | ns | ns |  |  |  |  |  |  |
| Cupressaceae (spring) | T-max | ns | ns | ns |  |  |  |  |  |  |  |
|  | T-min | ns | ns | ns |  |  |  |  |  |  |  |
| *Morus* | T-max | ns | -0.504** | ns | ns |  |  |  |  |  |  |
|  | T-min | -0.363* | -0.390* | ns | ns |  |  |  |  |  |  |
| *Platanus* | T-max | ns | ns | ns | ns |  |  |  |  |  |  |
|  | T-min | ns | ns | ns | ns |  |  |  |  |  |  |
| Poaceae | T-max | ns | ns | ns | ns | ns | ns |  |  |  |  |
|  | T-min | ns | ns | ns | ns | ns | ns |  |  |  |  |
| *Quercus* | T-max | -0.515** | ns | ns | ns |  |  |  |  |  |  |
|  | T-min | -0.359* | ns | ns | ns |  |  |  |  |  |  |
| *Ulmus* (spring) | T-max | ns | ns | ns |  |  |  |  |  |  |  |
|  | T-min | ns | ns | ns |  |  |  |  |  |  |  |
| *Ulmus* (fall) | T-max | ns | ns | 0.398* | ns | ns | ns | ns | ns | ns |  |
|  | T-min | ns | ns | ns | ns | ns | 0.441* | ns | ns | 0.488* |  |

**Supplementary Table 6**. Spearman Correlation of pollen season variables with total monthly precipitation. Statistical significance is indicated by *p <0.05; ** p <0.01; ***p <0.001. Only pollen taxa and pollen season variables with significant correlations are shown.

|  | Start Date | End Date | Peak Date | Peak Concentration |
| --- | --- | --- | --- | --- |
| *Ambrosia* |  |  | Apr: -0.345* | Sep: 0.367* |
| Poaceae |  | Feb: -0.427*  Sep: 0.415* | Apr: 0.556*** |  |
| *Ulmus* (fall) | Jan: 0.524** |  | Aug: -0.415* |  |

**
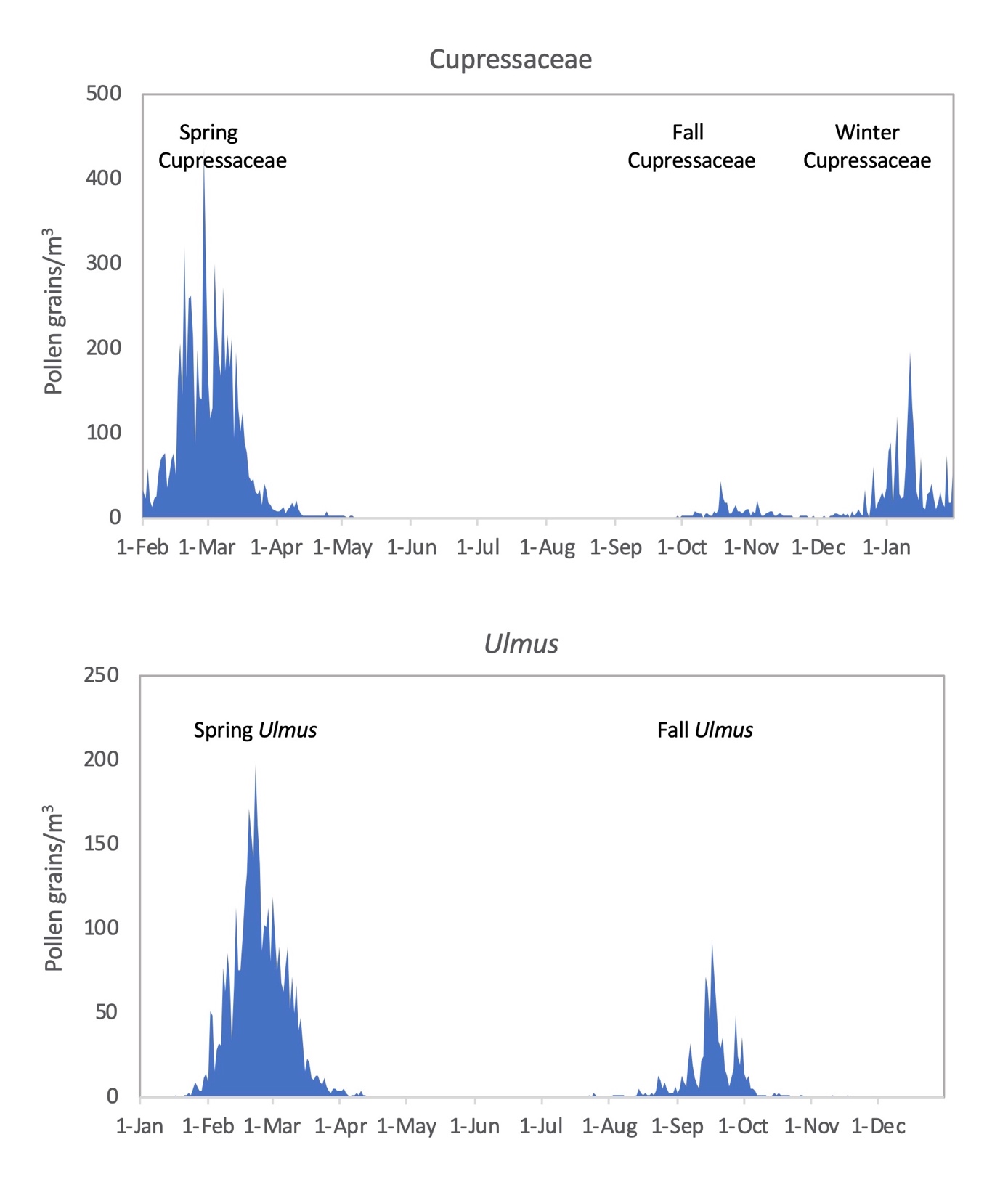
**

**Supplementary Figure 1**. Average daily concentrations of airborne Cupressaceae pollen and *Ulmus* pollen in Tulsa Oklahoma. The Cupressaceae graph shows the daily concentrations averaged over 34 years for spring Cupressaceae pollen (mainly *Juniperus virginiana*), fall Cupressaceae pollen (*J. pinchotii*), and winter Cupressaceae pollen (*J. ashei*). The *Ulmus* graph shows the daily concentrations averaged over 25 years for spring pollinating *Ulmus* species and fall pollinating *Ulmus* species.
